# *Reflexicalyptrales* ordo nov., a distinct *Agaricomycetes* lineage discovered from orchid mycorrhizae

**DOI:** 10.3897/imafungus.17.201939

**Published:** 2026-09-11

**Authors:** Akihiko Kinoshita, Shohei Fujimori, Kensuke Seto, Yuki Ogura-Tsujita, Ayako Maeda, Stephan W. Gale, Junichi P. Abe, Yousuke Degawa, Tomohisa Yukawa

**Affiliations:** 1 Kyushu Research Center, Forestry and Forest Products Research Institute, 4-11-16 Kurokami, Kumamoto 860-0862, Japan Kyushu Research Center, Forestry and Forest Products Research Institute Kumamoto Japan; 2 Graduate School of Life and Environmental Sciences, University of Tsukuba, 1-1-1 Tennodai, Tsukuba, Ibaraki, 305-8572, Japan Graduate School of Life and Environmental Sciences, University of Tsukuba Tsukuba Japan; 3 Sugadaira Research Station, Mountain Science Center, University of Tsukuba, 1278-294, Sugadaira-Kogen, Ueda, Nagano 386-2204, Japan Sugadaira Research Station, Mountain Science Center, University of Tsukuba Nagano Japan; 4 Institute for Multidisciplinary Sciences (IMS), Yokohama National University, 79-7, Tokiwadai, Hodogawa, Yokohama, Kanagawa 240-8501, Japan Institute for Multidisciplinary Sciences (IMS), Yokohama National University Yokohama Japan; 5 Faculty of Agriculture, Saga University, Honjo-machi 1, Saga 840-8502, Japan Faculty of Agriculture, Saga University Saga Japan; 6 The Kochi Prefectural Makino Botanical Garden, Godaisan 4200-6, Kochi 781-8125, Japan The Kochi Prefectural Makino Botanical Garden Kochi Japan; 7 Kadoorie Farm and Botanic Garden, Lam Kam Road, Tai Po, New Territories, Hong Kong S.A.R., China Kadoorie Farm and Botanic Garden Hong Kong S.A.R. China; 8 Tsukuba Botanical Garden, National Museum of Nature and Science, 4-1-1 Amakubo, Tsukuba, Ibaraki 305-0005, Japan Tsukuba Botanical Garden, National Museum of Nature and Science Tsukuba Japan

**Keywords:** *
Basidiomycota
*, dark matter fungi, environmental samples, new taxa, phylogeny, septal pore cap, ultrastructure

## Abstract

Uncovering the identity of “dark fungi” is crucial for understanding fungal taxonomy, diversity, and phylogenetic relationships. Previous exploratory analysis of the mycorrhizal fungi of the partially mycoheterotrophic orchid *Nervilia nipponica* revealed an association with a particular group of *Agaricomycetes*, tentatively designated “unidentified *Agaricomycetes* X” (uAX). Two culture strains were successfully isolated from the mycorrhiza, which enabled the phylogenetic position of this fungus to be determined using ultrastructural and multigene phylogenetic analyses. Transmission electron microscopy observations of the two cultures of uAX revealed that the dolipore septum is associated with perforate septal pore caps (SPCs), the margins of which are reflexed outwards. This combination of characters has not previously been reported in any order of *Basidiomycota*. Combined phylogenetic analyses based on sequence data from four loci [5.8S and 28S ribosomal DNA (rDNA), translation elongation factor 1-α, and RNA polymerase II second large subunit] indicate that uAX is an early-diverging clade of *Agaricomycetes* that cannot be accommodated in any known order of *Basidiomycota*. The name *Reflexicalyptrales* is proposed here for this new order of *Basidiomycota*. Furthermore, BLAST searches of the internal transcribed spacers of nuclear ribosomal DNA (nrDNA) and phylogenetic analyses reveal that closely related taxa have previously been detected in soil, leaf litter, and living root samples collected at various locations worldwide. This suggests that the group is ubiquitously distributed and probably exhibits a saprotrophic lifestyle

## Introduction

Fungi are one of the most diverse groups of eukaryotes, and they play critical roles in terrestrial and aquatic ecosystems through their parasitic, mutualistic, and saprotrophic modes of nutrition (McLaughlin and Spatafora 2014). However, Hawksworth and Lücking (2017) estimated that less than 8% of fungal taxa have been described. The advent of high-throughput sequencing technologies offers significant advances in uncovering and classifying fungal diversity (Tedersoo et al. 2014; Baldrian et al. 2021). For example, Tedersoo et al. (2017) uncovered more than 40 potential novel fungal groups at the order and class levels from global soil samples, of which nine lineages were placed in *Ascomycota* and seven in *Basidiomycota*. However, these novel fungal lineages—“dark taxa” or “dark matter fungi” (Grossart et al. 2016; Ryberg and Nilsson 2018)—cannot yet be published as new taxa because the rules of the International Code of Nomenclature for algae, fungi, and plants (ICN) stipulate the necessity of physical specimens from which morphological characters can be discerned (Hawksworth et al. 2016; Hawksworth and Lücking 2017; Hongsanan et al. 2018).

Nomura et al. (2013) revealed the occurrence of a single novel lineage comprising four internal transcribed spacer (ITS) sequence variants of *Agaricomycetes (Basidiomycota)* as the predominant mycorrhizal partner of *Nervilia nipponica* Makino, a terrestrial orchid species distributed in Japan and Korea that has since been shown to be partially mycoheterotrophic (Gale et al. 2010, 2021; Eum et al. 2011). These fungal partners were tentatively designated “unidentified *Agaricomycetes* X” [hereafter, uAX (types I–IV)]. Phylogenetic analyses using a combined dataset consisting of 5.8S ribosomal DNA (rDNA), ITS2, and partial 28S rDNA further indicated that its phylogenetic placement was ambiguous because of weak support among several major related *Agaricomycetes* lineages. In this study, two culture strains were successfully isolated from newly sampled mycorrhiza. These strains were used to clarify the taxonomic placement of uAX by conducting more in-depth phylogenetic analyses. An expanded dataset was used that included several recently described *Basidiomycota* orders based on an updated classification of *Agaricomycotina* (He et al. 2019; Varga et al. 2019; James et al. 2020) and four genetic regions, *viz*., 5.8S rDNA, 28S rDNA, translation elongation factor 1-α (*tef1-α*), and RNA polymerase II second largest subunit (*rpb2*). Transmission electron microscopy (TEM) observations were also conducted using the established strains to clarify the dolipore septum structure of the fungus—more specifically, the microanatomy of the pore cap and surrounding septal pore, an important character for *Basidiomycota* classification (van Driel et al. 2009).

## Materials and methods

### Isolation of fungi from *Nervilia nipponica* mycorrhizae

Whole plants of *N. nipponica* were collected with adhering soil from Nankoku-shi, Kochi Prefecture, Japan, in June 2013. Underground parts were washed thoroughly under running tap water to remove soil particles and checked for mycorrhizal colonization. Mycorrhizal parts of three individuals were cut into pieces 1–2 cm long, from which longitudinal sections including the stele (200–500 μm in length) were prepared. Pelotons visualized in the sections were observed under a compound microscope (BX51, Olympus, Tokyo, Japan). After the epidermis was removed from the sections, they were transferred to distilled water and incubated at 4 °C for 1 h, dried on sterilized filter paper for 1 min, and placed in 1/6 nutrient dextrose yeast agar (1/6 NDY) medium (Masuhara and Katsuya 1992). The dishes were incubated for 22–36 days at 4 °C in the dark. To establish the fungal strain in pure culture, a single hypha was isolated from each section and incubated on a plate of potato agar [PA; 1000 mL of filtrate from 200 g of boiled potato and 20 g of agar (Wako Pure Chemical Industries, Ltd., Osaka, Japan)] at 17 °C in the dark. Established cultures were maintained on PA at 17 °C in the dark and transferred to fresh medium every month. Two strains, T469 and T470, were obtained, both of which were incubated on potato dextrose agar [PDA (BD Difco, Franklin Lakes, New Jersey, USA)] and deposited in the Research Center of Genetic Resources, NARO (hereafter, T469 and T470 are designated MAFF425704 and MAFF425705, respectively).

### Observations of dolipore septum ultrastructure

Hyphae of MAFF425704 growing on cellophane over PA were embedded in 1.5% water agar, from which solidified pieces (2–5 mm^2^) containing hyphae were cut. For fixation, these agar blocks were placed in 2% paraformaldehyde and 2.5% glutaraldehyde in 25 mM phosphate buffer (PB) adjusted to pH 7.0 at room temperature for 2 h. After being washed three times in 25 mM PB, the agar blocks were postfixed with 1% osmium tetroxide in 25 mM PB at 5 °C overnight. The blocks were dehydrated in an ethanol series (10%, 30%, 50%, 70%, and 90% for 15 min per step, followed by 95% once and 100% twice for 20 min each time) and embedded in Agar Low Viscosity Resin (Agar Scientific, Essex, UK). Ultrathin sections (50–100 nm) were prepared with a diamond knife set on an RMC MT-XL ultramicrotome (RMC Products, Tucson, Arizona, USA). Sections were stained with 2% (w/v) uranyl acetate and lead citrate (Reynolds 1963) and observed with a transmission electron microscope (HT7700, Hitachi, Tokyo, Japan) at an accelerating voltage of 80 kV. A three-dimensional model of the septal pore cap of strain MAFF425704 was reconstructed from the serial TEM section images using paper clay. The overall structure, including the thickness of the pore cap and the size and distribution of perforations, was determined based on the TEM images.

### Physiological characterization

To investigate the optimal temperature for mycelial growth, a single 4-mm agar block of each isolate (MAFF425704 and MAFF425705) was cut from a precultured colony and grown on PDA for six days at 20 °C in the dark. The blocks were subsequently placed at the center of new PDA medium (9 cm in diameter) and incubated for three days at 17 °C in the dark. These plates were then incubated at one of seven temperatures (5 °C, 10 °C, 15 °C, 20 °C, 25 °C, 30 °C, and 35 °C) in the dark. For each incubation temperature, the position of the edge of the mycelial colony was observed and recorded every 24 or 72 h for one month, by which time the colonies had grown to the edge of the inner plate. The growth rate was calculated from the average of 12 replicates. A one-way analysis of variance (ANOVA) followed by a Tukey–Kramer honest significant difference (HSD) test was performed to compare growth rates across temperatures using R 4.3.2 (R Core Team).

To observe the number of nuclei within the hyphae, mycelia from pure cultures on PDA were fluorescently stained after plating onto glass and mounted in 10 ppm 4,6-diamidino-2-phenylindole (DAPI) and 200 mg/mL Calcofluor White in the dark at 25 °C for 10 min. Nuclei were observed under a fluorescence microscope (Axioscope 5, Carl Zeiss, Switzerland) using an excitation filter (330–385 nm) and absorbing filter (420 nm).

Morphological characteristics of the fungal colonies on different culture media were examined. A single 4-mm agar block, precultured on PDA in the dark for six days, was placed on each of the following media: CMA, OA, 1/6 NDY supplemented with 100 ppm streptomycin and 50 ppm tetracycline, PA, PDA, and MA; cultures were incubated at 20 °C in the dark. After 14 days, colony color and morphological characters of aerial hyphae were observed.

Fruitbody induction was further examined. Mycelial disks (5 mm in diameter) taken from the edges of 20-day-old colonies on PDA culture were inoculated into sterilized cedar (*Cryptomeria japonica*) and evergreen Japanese oak (*Quercus* spp.) sawdust in Petri dishes 9 cm in diameter. The moisture content of the sawdust substrate was adjusted to 65% by mixing sawdust and bran at a ratio of 3:1. These dishes were incubated at 22 °C in the dark for 50 days, and the development of the colonies was observed under a stereomicroscope, optical microscope, and scanning electron microscope (Miniscope TM-1000, Hitachi, Tokyo, Japan). To stimulate fruitbody production, portions of the culture medium 2 cm in diameter were transferred to fresh sawdust medium in 650 mL bottles and 1.0 kg plastic bags. After 50 days, the culture medium was flooded with tap water for 1 h, the water was decanted, and the medium was irradiated and then cultured again at 20 °C for 30 days.

### PCR amplification and sequencing

In *Basidiomycota*, higher-level taxonomy at the order or family level has often been resolved by multigene phylogenetic approaches (e.g., Binder et al. 2010; Hodkinson et al. 2014; Sjökvist et al. 2014; Johnston et al. 2019). Therefore, to provide greater resolving power in pinpointing the phylogenetic position of uAX, two additional commonly used loci, *rpb2* and *tef1-α*, were sequenced (Matheny et al. 2007; Binder et al. 2010).

PCR amplification was conducted using the four types of uAX DNA samples previously analyzed by Nomura et al. (2013) from *N. nipponica* mycorrhiza (type I: M293; type II: M1051; type III: M38; type IV: M1277; Suppl. material 6: table S1), with the primer pairs fRPB2-5F/bRPB2-7R2 for *rpb2* (Liu et al. 1999; Matheny et al. 2007) and EF1-983F/EF1-1567R for *tef1-α* (Rehner and Buckley 2005). PCR products that were difficult to sequence directly were cloned using the pGEM-T Vector System II (Promega, Madison, Wisconsin, USA). Twelve positive clones per PCR product were randomly chosen for sequencing. PCR products were purified using ExoSAP-IT (Affymetrix, Santa Clara, CA, USA) and sequenced on an ABI3130xl automated capillary sequencer (Applied Biosystems, Foster, CA, USA) with the BigDye Terminator 3.1 Cycle Sequencing Kit (Applied Biosystems, Foster, CA, USA), following the manufacturer’s instructions. The following internal primers were used for sequencing in addition to the PCR primers for *rpb2*: bRPB2-6F and gRPB2-6R (Liu et al. 1999; Matheny 2005).

To identify the two established fungal isolates (MAFF425704 and MAFF425705), PCR amplification and sequencing of ITS and 28S rDNA were performed using the ITS5/ITS4 and NL1/NL4 primer pairs, respectively (White et al. 1990). Additionally, sequences of the *tef1-α* and *rpb2* regions for MAFF425704 were obtained by the aforementioned procedure.

### Sequence alignment and phylogenetic analyses

To determine the phylogenetic placement of uAX, sequence data representative of the main *Basidiomycota* lineages were selected. Sequences of 28S and 5.8S rDNA, *rpb2*, and *tef1-α* were obtained mainly from the Assembling the Fungal Tree of Life (AFToL) database (Suppl. material 6: table S1). Phylogenetic analyses were performed using a combined dataset of 28S, 5.8S, *rpb2*, and *tef1-α* sequences (four-locus dataset), as well as each locus independently, under maximum likelihood (ML) criteria and Bayesian inference.

For the four-locus dataset, two or three representative sequences of all existing *Basidiomycota* orders (He et al. 2019; Varga et al. 2019; James et al. 2020) were downloaded from the International Nucleotide Sequence Database (INSD, DDBJ/EMBL/GenBank) (Suppl. material 6: table S1). In particular, all families of *Cantharellales* and *Phallomycetidae* (*Gomphales*, *Hysterangiales*, and *Geastrales*) were included because the results of Nomura et al. (2013) suggested that these groups had a close affinity to uAX. Three sequences of *Ascomycota* (*Morchella galilaea*, *Saccharomyces cerevisiae*, and *Tuber magnatum*) were chosen as outgroups. Each dataset (28S, 5.8S, *rpb2*, and *tef1-α*) was aligned separately using the MAFFT 7 online server (https://mafft.cbrc.jp/alignment/software/) (Katoh and Standley 2013), with poorly aligned sites identified using Gblocks 0.91b (Castresana 2000). Individual alignments for the four regions were then combined into a single dataset.

Maximum likelihood analyses were performed using IQ-TREE 2.3.6 (Minh et al. 2020). The TIM3+I+G4, TIM3u+G4, TIM3+F+I+G4, and GTR+I+G4 models of nucleotide evolution were selected as the best substitution models for 28S, 5.8S, *tef1-α*, and *rpb2*, respectively, following evaluation with the Bayesian information criterion (BIC) by ModelFinder (Kalyaanamoorthy et al. 2017) implemented in IQ-TREE. Shimodaira–Hasegawa approximate likelihood ratio test (SH-aLRT) and ultrafast bootstrap values were obtained with 10,000 replicates each and used as measures of branch support. The final tree was visualized with the Interactive Tree Of Life (iTOL) (Letunic and Bork 2006).

Bayesian analyses were conducted in MrBayes 3.2.7a (Ronquist et al. 2012). The GTR+G model was applied for 28S, GTR+I+G for 5.8S and *rpb2*, and SYM+I+G for *tef1-α*. State frequencies, substitution matrices, shape parameters, and the proportion of invariable sites were estimated for each locus. Four independent Markov chain Monte Carlo (MCMC) chains were run, sampling every 1000^th^ tree until the average standard deviation of split frequencies (ASDSF) became <0.01. The log files of MrBayes were further analyzed using Tracer 1.7 (Rambaut et al. 2018) to check the effective sample sizes, which were always >200, indicating sufficient independent sampling to estimate the posterior distribution of each parameter. The first 10% of the sampled trees were discarded as burn-in. The remaining trees were used to construct a 50% majority-rule consensus tree, which was visualized with iTOL.

The five closest matching sequences for each of the four types of uAX were retrieved by BLAST searches (Altschul et al. 1994) using the 28S, *rpb2*, and *tef1-α* loci. These sequences were then added to each dataset for further analysis (accessed on 14 October 2025; Suppl. material 7: table S2). In addition, four additional major novel lineages within *Agaricomycetes* (GS28, GS29, GS30, and branch 5) were included in the 28S dataset; these lineages had been identified from soil DNA samples using metabarcoding and phylogenetic approaches (Suppl. material 8: table S3; Tedersoo et al. 2017). For the protein-coding genes (*rpb2* and *tef1-α*), nucleotide sequences were translated into amino acid sequences to confirm correct alignment and minimize potential codon misalignment. Amino acid alignments were then used to guide the corresponding nucleotide alignments. Introns were excluded for both loci. Gblocks trimming was not applied to *rpb2* and *tef1-α* to preserve codon positions for partitioned codon-based phylogenetic analyses. Partitioned evolutionary models were applied to *rpb2* and *tef1-α* by separating the first, second, and third codon positions of each gene for ML analyses (Suppl. material 9: table S4). In Bayesian analysis, codon-partitioned analyses were initially performed for *tef1-α* and *rpb2*; however, because of poor convergence of the MCMC chains, the final Bayesian analyses were conducted using locus-based partitions.

The nuclear ribosomal DNA (nrDNA) ITS region, a fungal DNA barcoding region, is suitable for grouping samples based on sequence similarity and inferring the ecology and distribution of target fungal taxa (Nilsson et al. 2019). In assigning taxonomic groups, a threshold value of 90% sequence identity is used for genus, 85% for family, 80% for order, and 75% for class (Tedersoo et al. 2014; Nilsson et al. 2019). ITS divergence was investigated within the four uAX types, as well as between uAX and the eight sequences of *Tremellodendropsidales*, the most likely sister lineage of uAX. These eight sequences included those from *Tremellodendropsis tuberosa* (KU900857–KU900859 and KU900861; Berbee et al. 2016) and environmental sequences belonging to *Tremellodendropsidales* (KY687729 and KY687667; Tedersoo et al. 2017; JX316510 and JX316518; Truong et al. 2017).

To explore the ecology and distribution of close relatives of uAX, massBLASTer analysis was conducted on ITS sequences of the four types of uAX in the UNITE database, including INSD and high-throughput sequencing (HTS) data (Abarenkov et al. 2023). Data for 30 representative sequences for each species hypothesis (SH) were selected from the BLAST results for each of the four types of uAX, and information on their distribution, origin, and other geographic characteristics was downloaded (accessed on 15 October 2025). Preliminary phylogenetic analyses using *Tremellodendropsidales*, *Trechisporales*, or both as outgroups indicated that high genetic divergence between ingroup and outgroup taxa resulted in low resolution in terms of both tree length and branch support. Therefore, unrooted phylogenetic analysis was performed with the retrieved sequences (Suppl. material 10: table S5). All alignment files used in this study have been deposited in Figshare (http://doi.org/10.6084/m9.figshare.32592672) and are also provided as Suppl. materials 11–15.

## Results

### Identification of two strains isolated from *N. nipponica* mycorrhizae

**Figure 1.**
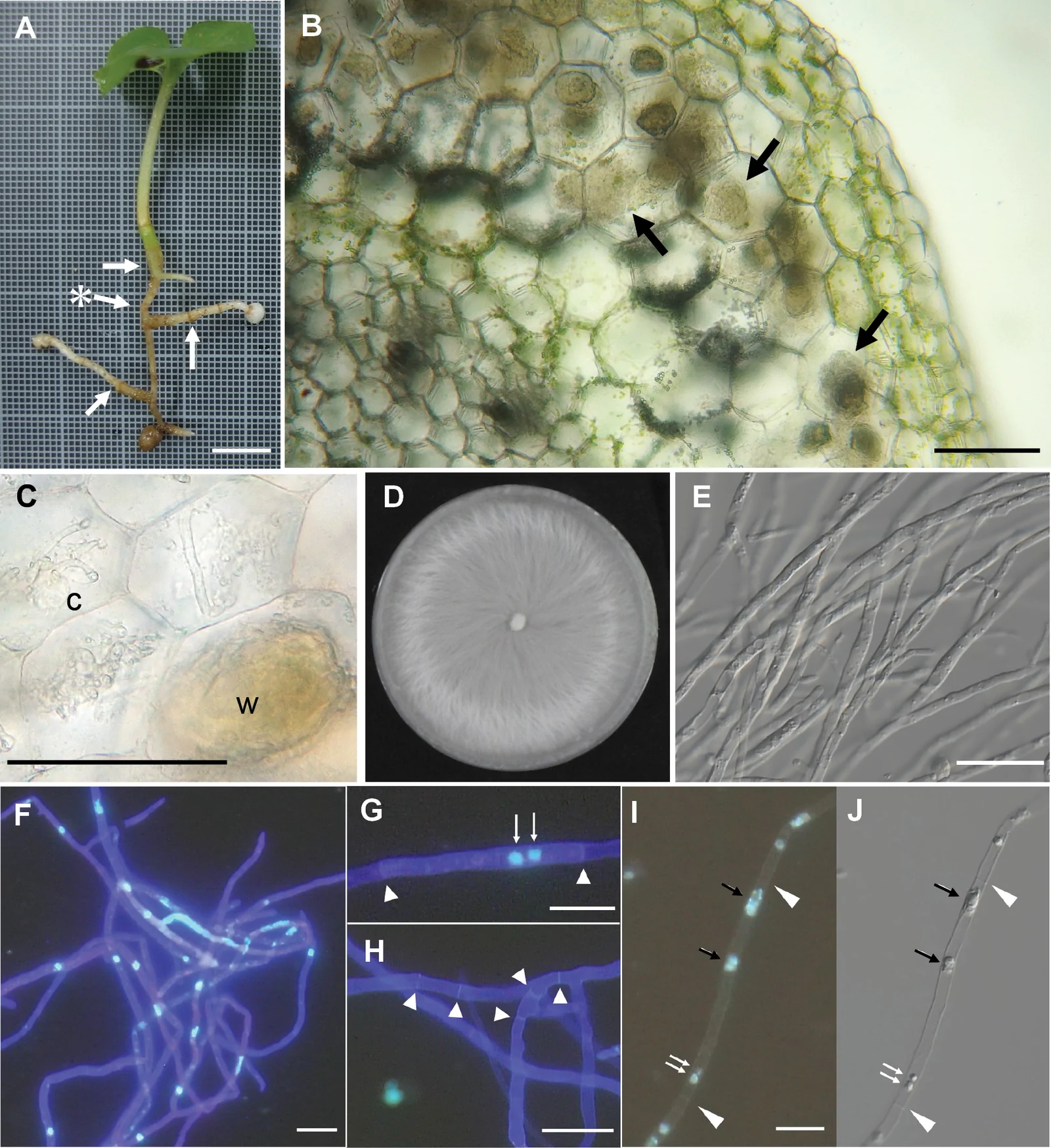
**A**. Host plant *Nervilia nipponica*; the arrow indicates the root system where mycorrhizae formed by *Reflexicalyptra japonica* were detected, and the asterisk (*) indicates the mycorrhizae from which the transverse section (**B**) was prepared. **B**. Transverse section of *N. nipponica* mycorrhiza; arrows indicate fungal structures formed by *R. japonica*. **C**. Hyphal structures formed within cortical cells; c, coralloid hyphae; w, peloton-like hyphae. **D**. Mycelial colony of MAFF425704 on PDA. **E**. Vegetative hyphae observed under phase-contrast microscopy. **F–I**. Fluorescence microscopy images of hyphae of MAFF425704 on PDA stained with DAPI and Calcofluor White; white arrows and arrowheads indicate putative nuclei and septa, respectively. **J**. Phase-contrast image corresponding to **I**; black arrows indicate vacuole-like structures corresponding to diffuse DAPI-stained regions (**I**, **J**). Scale bars: 1 cm (**A**); 100 μm (**B**); 10 μm (**E–I**).

Two culture strains (MAFF425704 and MAFF425705) were successfully isolated from the mycorrhiza of *N. nipponica* (Fig. 1). Both strains were obtained from the base of the inflorescence that developed from the underground tuber of a single individual; other plant parts failed to yield fungal material. The ITS and 28S rDNA sequences of the two strains were 100% identical to each other and matched uAX type I (Nomura et al. 2013) with 100% similarity.

### Phylogenetic placement of uAX in *Basidiomycota*

The aligned sequence lengths, as well as the numbers of OTUs, phylogenetically informative sites, variable sites, and non-variable sites in the four-locus combined and individual datasets (28S, 5.8S, *rpb2*, and *tef1-α*) used for phylogenetic analyses, are shown in Suppl. material 9: table S4.

**Figure 2.**
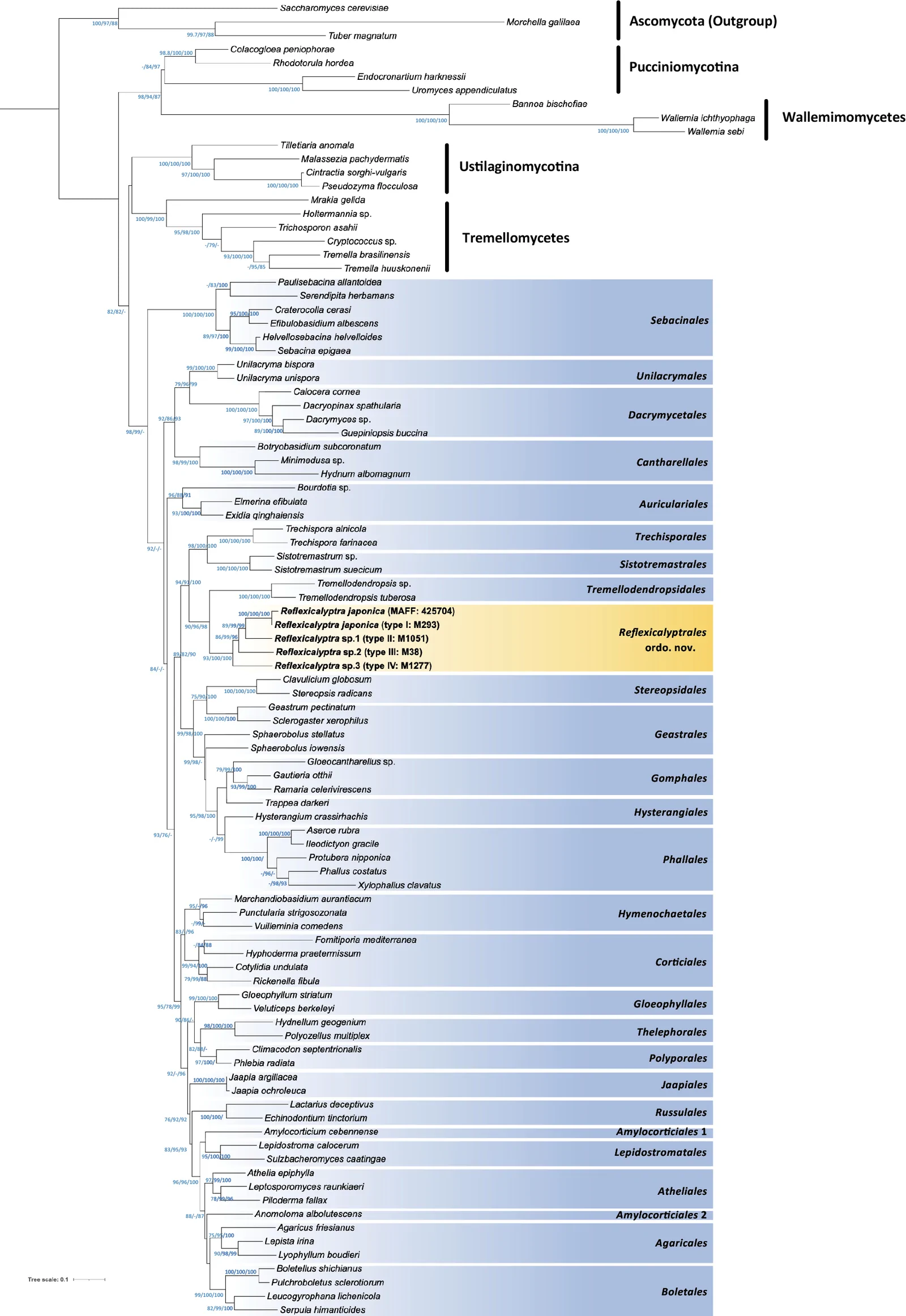
Maximum likelihood phylogeny inferred from a combined dataset of four loci (28S + 5.8S + *tef1-α* + *rpb2*). ML aLRT/ML ultrafast bootstrap support values >75% and Bayesian posterior probabilities >80% are shown on individual branches. *Reflexicalyptrales* is highlighted in yellow.

In the four-locus phylogeny, *Sebacinales* branched after *Tremellomycetes* and prior to *Dacrymycetales* and *Unilacrymales* in the ML analysis (ML aLRT = 98%/ultrafast bootstrap value = 99%), whereas in the Bayesian inference (BI) tree, *Sebacinales* was placed outside *Dacrymycetales* and *Unilacrymales* with maximum support (Bayesian posterior probabilities = 100) (Fig. 2). The monophyly of the four types of uAX was strongly supported by ML aLRT, ultrafast bootstrap values, and Bayesian posterior probabilities (93/100/100) (Fig. 2). Individual phylogenetic analyses of each of the three regions (28S, *tef1-α*, and *rpb2*) exhibited some discrepancies in branching order among *Basidiomycota* orders, especially in the *tef1-α* dataset (Suppl. materials 1–3: figs S1–S3). Nevertheless, the monophyly of the four uAX lineages was strongly supported (28S: 100/100/100; *rpb2*: 100/100/96; and *tef1-α*: 97/99/100). Furthermore, whereas ITS sequence similarity among the four types of uAX was 89–96%, similarity between each uAX type and the eight *Tremellodendropsidales* sequences ranged from 63% to 76%. In the combined analysis of the four loci and the 28S rDNA dataset, which had the densest taxon sampling, the four uAX types exhibited a sister relationship with the *Tremellodendropsidales* clade with strong support (four loci: 90/96/98; 28S rDNA: 88/97/92) (Fig. 2; Suppl. material 1: fig. S1). Together, these two clades formed a sister relationship with *Trechisporales* and *Sistotremastrales* in the four-locus phylogeny (Fig. 2; Suppl. material 1: fig. S1).

### Ecology and distribution inferred from environmental sequences

**Figure 3.**
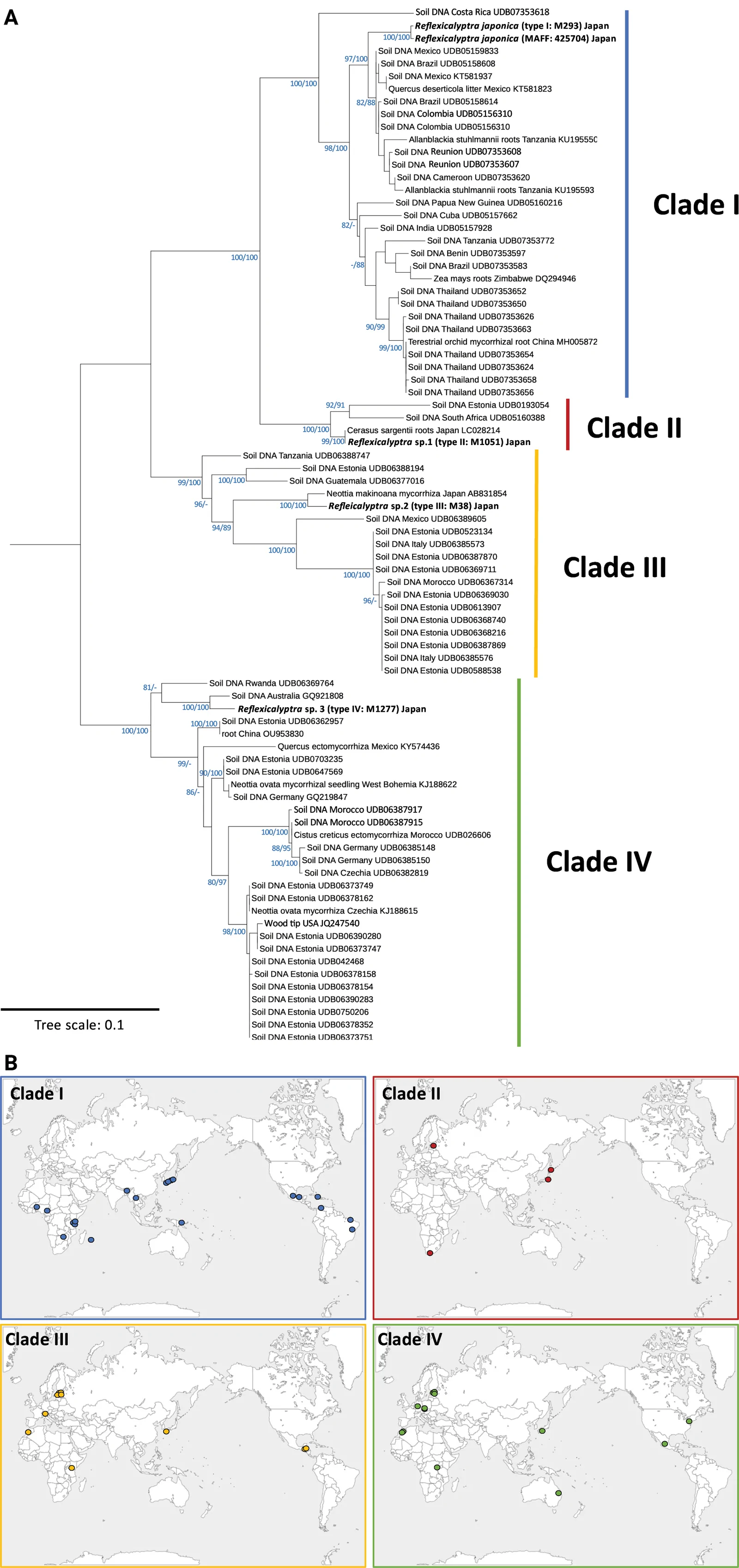
**A**. Unrooted maximum likelihood phylogeny based on the five ITS sequences of *Reflexicalyptrales* generated in this study and their homologous INSD, UNITE, and HTS sequences. ML aLRT/ML ultrafast bootstrap support values >75% and Bayesian posterior probabilities >80% are shown on individual branches. **B**. Maps indicating the source locations of the four *Reflexicalyptrales* clades recognized in the phylogenetic analyses.

Phylogenetic relationships inferred from the BLAST searches using ITS sequences from the four uAX types as references are shown in Fig. 3, and collection localities of the retrieved sequences are shown in Suppl. material 10: table S5. In the ITS phylogeny, four clades corresponding to each of the four uAX types were identified (Fig. 3A). The clade containing uAX type I mainly consisted of sequences derived from forest soils, as well as those isolated from *Zea mays* L. and *Allanblackia stuhlmannii* (Engl.) Engl. root samples and leaf litter (Fig. 3A; Suppl. material 10: table S5). This clade primarily comprised sequences from temperate to tropical biomes (Fig. 3B). In contrast, ITS sequences from uAX type II exhibited >99% similarity with a fungal isolate obtained from fine, live roots of *Cerasus sargentii* (Rehder) H.Ohba collected in Japan. This clade had the fewest OTUs but included sequences derived from forest soils collected in Estonia and South Africa (Fig. 3). The clade containing uAX type III comprised sequences from forest soils collected across Europe, Africa, and Central America, although the uAX type III sequences were sister to those derived from mycorrhizae of the Japanese endemic chlorophyllous orchid *Neottia makinoana* (Ohwi) Szlach. Finally, the clade containing uAX type IV included sequences collected globally from diverse substrates spanning forest and open woodland soils and leaf litter, as well as *Cistus* and *Quercus* ectomycorrhizae and orchid mycorrhizae.

### Morphological characters and ultrastructure

**Figure 4.**
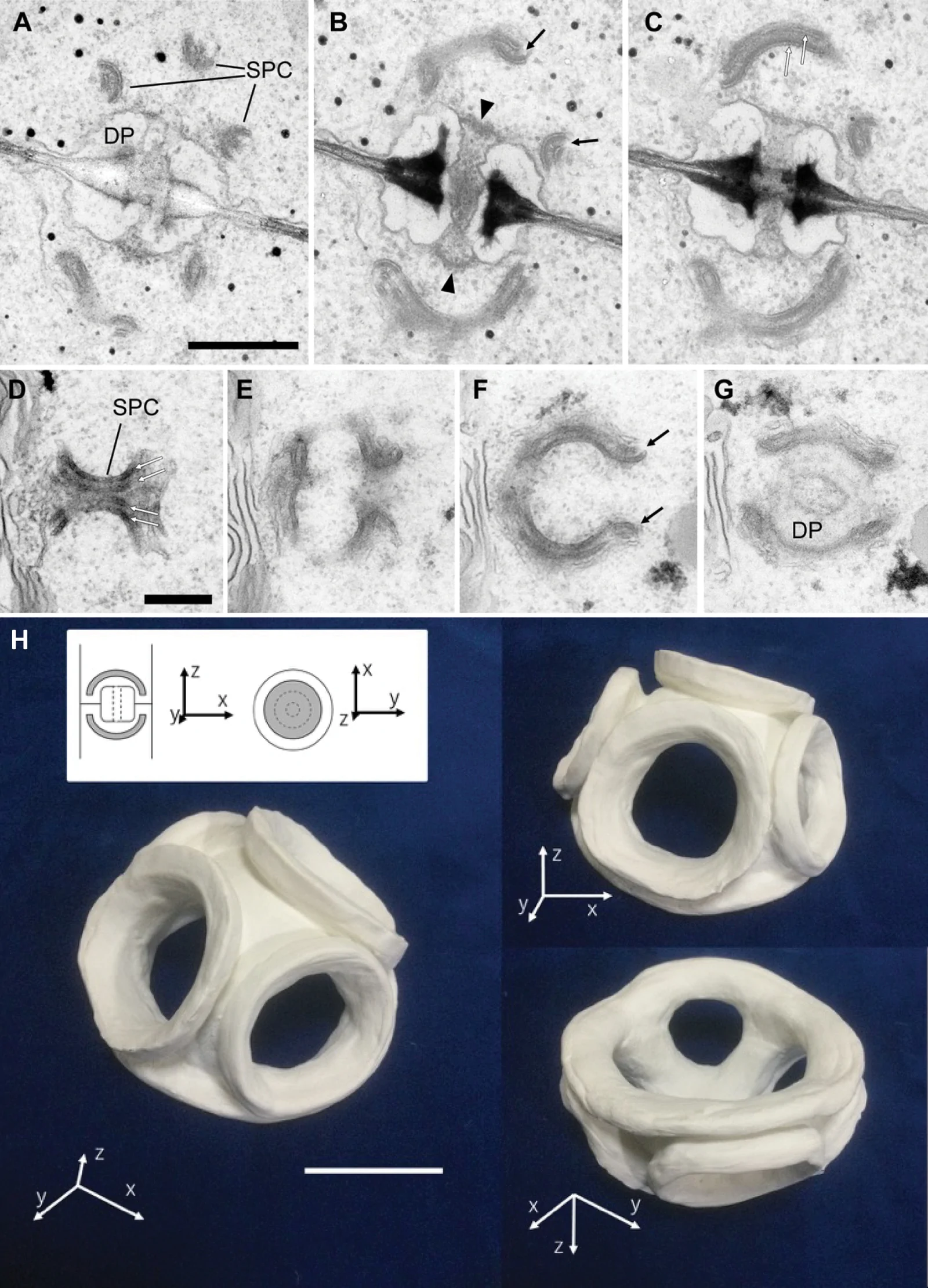
Ultrastructure of hyphal septa and morphology of *Reflexicalyptra japonica* strain MAFF425704. **A–C**. TEM images of longitudinal serial sections of a hyphal septum. **D–G**. TEM images of transverse serial sections of the hyphal septum. DP: dolipore; SPC: septal pore cap. Arrowheads in **B** indicate electron-dense occlusion at the orifice of the dolipore. Black arrows in **B** and **F** indicate bent regions of the SPC. White arrows in **C** and **D** indicate the outer layers of the SPC. Scale bars: 500 nm (**A**, for **A–C**); 200 nm (**D**, for **D–G**). **H**. Three-dimensional model of a septal pore cap made of paper clay and created from TEM serial section images of *R. japonica* strain MAFF425704. The thickness and perforation size of the 3D model were based on TEM observations. Scale bar: 5 cm.

Longitudinal and transverse serial sections revealed that MAFF425704 had a dolipore septum associated with multi-pored (perforate) septal pore caps (SPCs) (Fig. 4), the margins of which were bent outwards (black arrows in Fig. 4B, F). Perforations of the SPCs in MAFF425704 were approximately 300 nm in diameter. The SPCs ranged from 75 to 90 nm in thickness and were constructed of three layers: two outer layers consisting of electron-dense lines (white arrows in Fig. 4C, D) and an inner layer that formed a less electron-dense band.

### Physiology and culture characteristics

Both strains (MAFF425704 and MAFF425705) cultured on PDA developed well at 10–30 °C, with maximum growth rates observed at 25 °C. Growth terminated at 5 °C and 35 °C (Suppl. material 4: fig. S4). Despite attempts to induce fruitbody production in MAFF425704, these structures failed to develop during the experiments. Nevertheless, uAX formed more than 50 sclerotia-like structures in a Petri dish during incubation (Suppl. material 5: fig. S5). These sclerotia doubled in size, turned brown, and produced flower-like druse and prismatic structures on the surface.

## Discussion

Phylogenetic relationships among *Basidiomycota* orders derived from the four concatenated datasets were mostly consistent with previous results of comprehensive phylogenetic analyses of *Basidiomycota*, except for the splitting of *Amylocorticiales* (Fig. 2) (Nagy et al. 2016; He et al. 2019, 2024; Varga et al. 2019; James et al. 2020; Tedersoo et al. 2020; Li et al. 2021). Although the overall topology was largely congruent with previous studies, *Sebacinales* was placed after *Tremellales* and prior to *Dacrymycetales* and *Unilacrymales* in the ML four-locus phylogeny with high support, slightly differing from previously reported relationships (e.g., Weiß et al. 2016; He et al. 2019, 2024), whereas the BI analysis supported the conventional placement of *Sebacinales*. Such method-dependent topological shifts among early-diverging heterobasidiomycetous lineages are likely attributable to differences in taxon sampling, locus selection (e.g., concatenation of four specific loci), or analytical algorithms. Crucially, this localized topological variation among outer basal groups in the ML tree does not affect the stable placement and strongly supported independence of uAX within *Agaricomycetes* in both ML and BI analyses.

Phylogenetic analyses based on multiple loci clearly demonstrate that uAX represents a distinct monophyletic lineage within *Agaricomycetes* that does not belong to any previously established order. In the concatenated four-locus and 28S rDNA datasets, uAX consistently forms a strongly supported sister clade to *Tremellodendropsidales* (Fig. 2; Suppl. material 1: fig. S1), and together they form a sister group to *Trechisporales* and *Sistotremastrales*. Although sequence divergence in the ITS region at higher taxonomic levels should be interpreted with caution, the low sequence similarity (63–76%) between uAX and *Tremellodendropsidales* aligns well with the threshold (≤80%) generally accepted for order-level differentiation in fungi (Tedersoo et al. 2014; Nilsson et al. 2019).

TEM observations revealed that uAX exhibits a highly novel dolipore septum ultrastructure characterized by uniquely reflexed pore margins—a feature hitherto unreported among known *Basidiomycota* (Fig. 4). Fungal taxa with perforated SPCs are known only in several *Agaricomycetidae* orders, including some members of *Cantharellales* and *Phallales* (van Driel et al. 2009), but these lineages are phylogenetically isolated from uAX (Fig. 2). Among closely related lineages, *Tremellodendropsis tuberosa* (Grev.) D.A.Crawford in *Tremellodendropsidales* (Mushroom Observer 196677; Berbee et al. 2016) and *Subulicystidium longisporum* (Pat.) Parmasto in *Trechisporales* (Keller 1997) were observed to exhibit an imperforate SPC type, clearly distinguishable from uAX. Perforation of the SPCs in MAFF425704 was approximately 300 nm in diameter, which represents a relatively large opening otherwise known only in certain *Cantharellales* taxa, including species of *Ceratobasidiaceae*, *Sistotrema* spp., *Craterellus cornucopioides* (L.) Pers., *Cantharellus cibarius* Fr., *Thanatephorus fusisporus* (J.Schröt.) Hauerslev & P.Roberts, and *Clavulina rugosa* (Bull.) J.Schröt. (Yanaga 2015). Furthermore, the three-layered arrangement of SPCs observed in uAX is similar to that observed in *Ceratobasidiaceae* (Tu et al. 1977; Oberwinkler et al. 2013). In this regard, perforation characters are shared between uAX and several taxa of *Ceratobasidiaceae* in *Cantharellales*, but these two lineages are phylogenetically distant (Fig. 2). Consequently, uAX cannot be accommodated in any previously established order of *Basidiomycota*, as it is distinctly differentiated from its phylogenetically close relatives by SPC ultrastructure and from morphologically similar taxa by molecular phylogeny.

### Ecological role and environmental distribution

The ITS sequence retrieval and environmental phylogenetic analyses indicate that uAX fungi have a ubiquitous, worldwide distribution across tropical, temperate, and cold biomes (Fig. 3). Rather than being restricted to specific mycorrhizal hosts, uAX lineages associate with diverse substrates and plant roots, including non-mycorrhizal or arbuscular mycorrhizal hosts (*Zea mays* and *Cerasus sargentii*), ectomycorrhizae (*Quercus* and *Cistus*), and orchid species (*Neottia nipponica* and *N. makinoana*). These findings suggest that uAX fungi possess versatile ecological roles—likely acting as saprotrophs, endophytes, or opportunists—rather than obligate ectomycorrhizal symbionts. The physiological temperature optimum of 25 °C recorded for strain MAFF425704 aligns well with the thermal environment of its host *N. nipponica* (Gale et al. 2021) and corresponds with the predominantly temperate-to-tropical distribution of uAX type I sequences.

### Morphogenesis and crystalline secretions

In the culture experiments, uAX formed brown sclerotia-like structures covered with flower-like druse and prismatic crystals (Suppl. material 5: fig. S5). Similar structures have been observed in the wood-decaying *Trichaptum abietinum* (Dicks.) Ryvarden in *Hymenochaetales* (Connolly et al. 1996), the ascomycetous pathogenic fungus *Sclerotinia sclerotiorum* (Lib.) de Bary (Uloth et al. 2015), and *Rhizoctonia solani* J.G.Kühn in *Cantharellales* growing on deadwood and living stems (Yang et al. 1993). Given that these structures in other fungi are known to be formed of calcium oxalate (Gadd et al. 2014), it is highly likely that uAX produces similar substances. However, it remains unclear what biological or ecological advantages this confers for the growth, development, and survival of uAX.

### Taxonomy

Since all molecular and morphological evidence presented here demonstrates that uAX is not affiliated with any known order of *Basidiomycota*, a new fungal order is proposed as follows. The type species, for which cultured strains were successfully obtained, is further described.

#### Reflexicalyptrales

Taxon classificationAnimaliaReflexicalyptralesReflexicalyptraceae

A.Kinosh. S.Fujimori, K.Seto & T.Yukawa ord. nov.

8A9511D4-2F13-5F03-AEA8-AAB24726F948

MB 854332

##### Type family.

*Reflexicalyptraceae* A.Kinosh. S.Fujimori, K.Seto & T.Yukawa

##### Description.

A dolipore septum associated with perforate septal pore caps (SPCs), the margins of which are bent outwards and constructed of three layers: two outer layers consisting of two electron-dense lines and an inner layer forming a less electron-dense band.

##### Diagnosis.

Distinguished from other known orders in *Agaricomycetes* by combined phylogenetic analyses of four loci (5.8S, 28S, *tef1-α*, and *rpb2*) and by septal pore caps (SPCs) with outer margins curved back (away from the septum). Differs from the phylogenetically related orders *Tremellodendropsidales* and *Trechisporales* by having perforate SPCs, in contrast to the imperforate SPCs of these orders.

#### Reflexicalyptraceae

Taxon classificationAnimaliaReflexicalyptralesReflexicalyptraceae

A.Kinosh. S.Fujimori, K.Seto & T.Yukawa fam. nov.

97E6D477-74BA-5A61-B5D7-6E1D99E0B9B1

MB 854333

##### Type genus.

*Reflexicalyptra* A.Kinosh. S.Fujimori, K.Seto & T.Yukawa

##### Description.

Description as for *Reflexicalyptrales*. Hyphae mostly binucleate, rarely uninucleate, clamp connection absent.

#### Reflexicalyptra

Taxon classificationAnimaliaReflexicalyptralesReflexicalyptraceae

A.Kinosh. S.Fujimori, K.Seto & T.Yukawa gen. nov.

7BF656B4-396B-5B2D-8AA9-CA61D88244C2

MB 854334

##### Type species.

*Reflexicalyptra japonica*, A.Kinosh. S.Fujimori, K.Seto & T.Yukawa, sp. nov. in this paper.

##### Etymology.

“*Reflexi-*” (Latin) = reflexed and “calyptra” (Latin) = cap referring to the reflexed form of the septal pore cap.

##### Description.

A dolipore septum associated with perforate septal pore caps (SPCs), the margins of which were bent outwards. Perforation of the SPCs was approximately 300 nm in diam. The SPCs ranged from 75–90 nm in thickness, constructed of three layers: two outer layers consisting of two electron-dense lines and an inner layer that formed a less electron-dense band. Hyphae mostly binucleate, rarely uninucleate, clamp connection absent, hyaline, mostly thin-walled. Forming coralloid or peloton-like hyphal structures within epidermal to cortical cells of roots and basal stems of *Nervilia nipponica*.

##### Notes.

Based on the ITS phylogeny, this lineage is expected to be rich in species. At present, detailed morphological features have been characterized only for the type species, *Reflexicalyptra japonica* (type I). Because physical specimens and morphological data for types II–IV are currently unavailable, further generic subdivision cannot be evaluated. Therefore, *Reflexicalyptra* is conservatively proposed as a comprehensive genus encompassing all clades within this lineage (types I–IV).

#### Reflexicalyptra japonica

Taxon classificationAnimaliaReflexicalyptralesReflexicalyptraceae

A.Kinosh. S.Fujimori, K.Seto & T.Yukawa sp. nov.

13114CA2-2068-56A4-B90A-D5BF817647B8

MB 854335

Figs 1, 4

##### Typification.

• JAPAN• KOCHI Prefecture, Nankoku-shi, Kinugasa; 33.5521°N, 133.6193°E; alt. 86 m; under *Castanopsis cuspidata* (Thunb.) Schottky. dominated evergreen broad-leaved forest; The host plant *Nervilia nipponica* was collected by Ayako Maeda (11 June 2013); culture strains were isolated from the mycorrhizal sections by Shohei Fujimori (4 July 2013); MAFF425704 (**holotype**, culture preserved in a metabolically inactive state by cryopreservation). Ex-type culture: MAFF425705. GenBank accession Nos.: LC820841 (ITSrDNA), LC820842 (28S rDNA), LC820843 (*tef1-α*), LC820844 (*rpb2*).

##### Etymology.

“*japonica*” (Latin) referring to the type locality “Japan.”

##### Description.

On PDA, colonies becoming white and velutinous as aerial mycelium, the surface around the edge of colonies being pannose as aerial hyphae. On PA, colonies becoming white and sheet-like and tufted as few aerial hyphae. On OA, colonies becoming white and tufted as aerial mycelium. On CMA, colonies becoming white and sheet-like with an absence of aerial mycelia. On MA, colonies becoming white to cream and velutinous to leathery as aerial hyphae. On 1/6 NDY with antibiotics, colonies becoming white and sheet-like and tufted as few aerial hyphae. On Japanese cedar and oak medium, colonies aggregate and forming sclerotia with spiky cystidia; after 100 days, sclerotia growing to >1 mm in diam. and producing crystal-like structures on surface. On PDA at 25 °C, hyphal colony growing 3.5–4.0 mm/d. Growth terminating at 5 °C and <35 °C. Hyphae mostly binucleate, rarely uninucleate, 1.5–4.0 μm in diam., clamp connection absent, hyaline, mostly thin-walled. Forming coralloid or peloton-like hyphal structures within epidermal to cortical cells of roots and basal stems of *N. nipponica*. Coralloid hyphae hyaline, < 3 µm in diam. Peloton-like hyphae light brown to dark brown.

##### Habitat and distribution.

In association with subterranean parts of *Nervilia nipponica* in Japan.

## Conclusion

While the discovery of new fungal taxa often stems from cryptic lineages or specimens retrieved from previously unexplored areas (Tedersoo et al. 2017; Truong et al. 2017), the findings demonstrate that even well-studied or common environments can harbor previously unrecognized higher-level fungal lineages. This study confirms the discovery of an entirely new fungal lineage that occurs in a common environment in Japan and is ubiquitous worldwide, a conclusion corroborated by the successful acquisition of physical specimens that enabled morphological, physiological, and molecular documentation of key characters. This novel order of *Basidiomycota* is formally described here as *Reflexicalyptrales*, and the confirmed taxa (family, genus, and species) within it are enumerated. Further research is needed to clarify other aspects of physiology, ecology, and taxonomy.

## Supplementary Material

XML Treatment for Reflexicalyptrales

XML Treatment for Reflexicalyptraceae

XML Treatment for Reflexicalyptra

XML Treatment for Reflexicalyptra japonica

## Additional information

### Artificial Intelligence (AI) use

The authors accept full responsibility for the content of the manuscript, including the disclosure of any use of AI.

No AI tools were used in the preparation of this manuscript.

### Adherence to national and international regulations

All the fungal strains used in this study have been legally obtained, respecting the Convention on Biological Diversity (Rio Convention).

### Author ORCIDs

A. Kinoshita https://orcid.org/0000-0002-7288-9780

K. Seto https://orcid.org/0000-0003-0606-0736

Y. Ogura-Tsujita https://orcid.org/0000-0003-4877-4702

S.W. Gale https://orcid.org/0000-0002-2161-6694

J.P. Abe https://orcid.org/0000-0003-0338-4495

Y. Degawa https://orcid.org/0000-0002-5955-1187

T. Yukawa https://orcid.org/0000-0003-4186-2371

### Data availability

All of the data that support the findings of this study are available in the main text or Supplementary Information.

## Supplementary materials

Supplementary material 1 Supplementary figure S1 Data type pdf Explanation note **figure S1**. Maximum likelihood phylogeny inferred from 28S rDNA sequences. Branch support values >75% are shown on individual branches of the ML and Bayesian analyses (ML aLRT/ML ultrafast bootstrap support/Bayesian posterior probabilities). *Reflexicalyptrales* is highlighted in yellow. https://binary.pensoft.net/file/1770303This dataset is made available under the Open Database License (http://opendatacommons.org/licenses/odbl/1.0/). The Open Database License (ODbL) is a license agreement intended to allow users to freely share, modify, and use this Dataset while maintaining this same freedom for others, provided that the original source and author(s) are credited. Akihiko Kinoshita, Shohei Fujimori, Kensuke Seto, Yuki Ogura-Tsujita, Ayako Maeda, Stephan W. Gale, Junichi P. Abe, Yousuke Degawa, Tomohisa Yukawa

Supplementary material 2 Supplementary figure S2 Data type pdf Explanation note **figure S2**. Maximum likelihood phylogeny inferred from *rpb2* sequences. ML aLRT/ML ultrafast bootstrap support values >75% and Bayesian posterior probabilities >80% are shown on individual branches. *Reflexicalyptrales* is highlighted in yellow. https://binary.pensoft.net/file/1770304This dataset is made available under the Open Database License (http://opendatacommons.org/licenses/odbl/1.0/). The Open Database License (ODbL) is a license agreement intended to allow users to freely share, modify, and use this Dataset while maintaining this same freedom for others, provided that the original source and author(s) are credited. Akihiko Kinoshita, Shohei Fujimori, Kensuke Seto, Yuki Ogura-Tsujita, Ayako Maeda, Stephan W. Gale, Junichi P. Abe, Yousuke Degawa, Tomohisa Yukawa

Supplementary material 3 Supplementary figure S3 Data type pdf Explanation note **figure S3**. Maximum likelihood phylogeny inferred from *tef1-α* sequences. ML aLRT/ML ultrafast bootstrap support values >75% and Bayesian posterior probabilities >80% are shown on individual branches. *Reflexicalyptrales* is highlighted in yellow. https://binary.pensoft.net/file/1770305This dataset is made available under the Open Database License (http://opendatacommons.org/licenses/odbl/1.0/). The Open Database License (ODbL) is a license agreement intended to allow users to freely share, modify, and use this Dataset while maintaining this same freedom for others, provided that the original source and author(s) are credited. Akihiko Kinoshita, Shohei Fujimori, Kensuke Seto, Yuki Ogura-Tsujita, Ayako Maeda, Stephan W. Gale, Junichi P. Abe, Yousuke Degawa, Tomohisa Yukawa

Supplementary material 4 Supplementary figure S4 Data type pdf Explanation note **figure S4**. Comparison of mycelial growth of the two isolated strains, MAFF425704 (**A**) and MAFF425705 (**B**), at 5–35 °C. Different letters above the boxes indicate significant differences among temperatures according to the Tukey–Kramer HSD test (*P* < 0.01). https://binary.pensoft.net/file/1770306This dataset is made available under the Open Database License (http://opendatacommons.org/licenses/odbl/1.0/). The Open Database License (ODbL) is a license agreement intended to allow users to freely share, modify, and use this Dataset while maintaining this same freedom for others, provided that the original source and author(s) are credited. Akihiko Kinoshita, Shohei Fujimori, Kensuke Seto, Yuki Ogura-Tsujita, Ayako Maeda, Stephan W. Gale, Junichi P. Abe, Yousuke Degawa, Tomohisa Yukawa

Supplementary material 5 Supplementary figure S5 Data type pdf Explanation note **figure S5. A**: Early (**a**) and late-stage (**b**) sclerotia-like structures formed on oak sawdust medium. SEM images of early (**B**) and late (**C**, **D**) stages of sclerotia-like structures. **C** and **D** show druse and crystal structures on the surface of the mycelium. Scale bars = 1 mm (**A**) and 300 μm (**B–D**). https://binary.pensoft.net/file/1770307This dataset is made available under the Open Database License (http://opendatacommons.org/licenses/odbl/1.0/). The Open Database License (ODbL) is a license agreement intended to allow users to freely share, modify, and use this Dataset while maintaining this same freedom for others, provided that the original source and author(s) are credited. Akihiko Kinoshita, Shohei Fujimori, Kensuke Seto, Yuki Ogura-Tsujita, Ayako Maeda, Stephan W. Gale, Junichi P. Abe, Yousuke Degawa, Tomohisa Yukawa

Supplementary material 6 Supplementary table S1 Data type pdf Explanation note **table S1**. Species and Genbank accession numbers used for the phylogenetic analysis. https://binary.pensoft.net/file/1770308This dataset is made available under the Open Database License (http://opendatacommons.org/licenses/odbl/1.0/). The Open Database License (ODbL) is a license agreement intended to allow users to freely share, modify, and use this Dataset while maintaining this same freedom for others, provided that the original source and author(s) are credited. Akihiko Kinoshita, Shohei Fujimori, Kensuke Seto, Yuki Ogura-Tsujita, Ayako Maeda, Stephan W. Gale, Junichi P. Abe, Yousuke Degawa, Tomohisa Yukawa

Supplementary material 7 Supplementary table S2 Data type pdf Explanation note **table S2**. Top five BLAST hit sequences of 28S, rpb2 and tef1-α sequences of four types uAX (accessed 14 Oct 2025). https://binary.pensoft.net/file/1770309This dataset is made available under the Open Database License (http://opendatacommons.org/licenses/odbl/1.0/). The Open Database License (ODbL) is a license agreement intended to allow users to freely share, modify, and use this Dataset while maintaining this same freedom for others, provided that the original source and author(s) are credited. Akihiko Kinoshita, Shohei Fujimori, Kensuke Seto, Yuki Ogura-Tsujita, Ayako Maeda, Stephan W. Gale, Junichi P. Abe, Yousuke Degawa, Tomohisa Yukawa

Supplementary material 8 Supplementary table S3 Data type pdf Explanation note **table S3**. Accession and isolate numbers of four novel lineages identified by Tedersoo et al. (2017), which were added to the 28 rDNA phylogenetic analysis. https://binary.pensoft.net/file/1770310This dataset is made available under the Open Database License (http://opendatacommons.org/licenses/odbl/1.0/). The Open Database License (ODbL) is a license agreement intended to allow users to freely share, modify, and use this Dataset while maintaining this same freedom for others, provided that the original source and author(s) are credited. Akihiko Kinoshita, Shohei Fujimori, Kensuke Seto, Yuki Ogura-Tsujita, Ayako Maeda, Stephan W. Gale, Junichi P. Abe, Yousuke Degawa, Tomohisa Yukawa

Supplementary material 9 Supplementary table S4 Data type pdf Explanation note **table S4**. The number of OTUs, sequence length, conserved, parsimony informative and variable sites used for phylogenetic analysis. https://binary.pensoft.net/file/1770311This dataset is made available under the Open Database License (http://opendatacommons.org/licenses/odbl/1.0/). The Open Database License (ODbL) is a license agreement intended to allow users to freely share, modify, and use this Dataset while maintaining this same freedom for others, provided that the original source and author(s) are credited. Akihiko Kinoshita, Shohei Fujimori, Kensuke Seto, Yuki Ogura-Tsujita, Ayako Maeda, Stephan W. Gale, Junichi P. Abe, Yousuke Degawa, Tomohisa Yukawa

Supplementary material 10 Supplementary table S5 Data type pdf Explanation note **table S5**. BLAST results of the ITSrDNA for four types of uAX against UNITE, INSD, and HTS sequences (accessed 15 Oct 2025). https://binary.pensoft.net/file/1770312This dataset is made available under the Open Database License (http://opendatacommons.org/licenses/odbl/1.0/). The Open Database License (ODbL) is a license agreement intended to allow users to freely share, modify, and use this Dataset while maintaining this same freedom for others, provided that the original source and author(s) are credited. Akihiko Kinoshita, Shohei Fujimori, Kensuke Seto, Yuki Ogura-Tsujita, Ayako Maeda, Stephan W. Gale, Junichi P. Abe, Yousuke Degawa, Tomohisa Yukawa

Supplementary material 11 The concatenated alignment of four DNA loci (ITS, LSU, tef1, and rpb2) Data type fas https://binary.pensoft.net/file/1770313This dataset is made available under the Open Database License (http://opendatacommons.org/licenses/odbl/1.0/). The Open Database License (ODbL) is a license agreement intended to allow users to freely share, modify, and use this Dataset while maintaining this same freedom for others, provided that the original source and author(s) are credited. Akihiko Kinoshita, Shohei Fujimori, Kensuke Seto, Yuki Ogura-Tsujita, Ayako Maeda, Stephan W. Gale, Junichi P. Abe, Yousuke Degawa, Tomohisa Yukawa

Supplementary material 12 Multiple sequence alignment data for ITS Data type fas https://binary.pensoft.net/file/1770314This dataset is made available under the Open Database License (http://opendatacommons.org/licenses/odbl/1.0/). The Open Database License (ODbL) is a license agreement intended to allow users to freely share, modify, and use this Dataset while maintaining this same freedom for others, provided that the original source and author(s) are credited. Akihiko Kinoshita, Shohei Fujimori, Kensuke Seto, Yuki Ogura-Tsujita, Ayako Maeda, Stephan W. Gale, Junichi P. Abe, Yousuke Degawa, Tomohisa Yukawa

Supplementary material 13 Multiple sequence alignment data for LSU Data type fas https://binary.pensoft.net/file/1770315This dataset is made available under the Open Database License (http://opendatacommons.org/licenses/odbl/1.0/). The Open Database License (ODbL) is a license agreement intended to allow users to freely share, modify, and use this Dataset while maintaining this same freedom for others, provided that the original source and author(s) are credited. Akihiko Kinoshita, Shohei Fujimori, Kensuke Seto, Yuki Ogura-Tsujita, Ayako Maeda, Stephan W. Gale, Junichi P. Abe, Yousuke Degawa, Tomohisa Yukawa

Supplementary material 14 Multiple sequence alignment data for tef1 Data type fas https://binary.pensoft.net/file/1770316This dataset is made available under the Open Database License (http://opendatacommons.org/licenses/odbl/1.0/). The Open Database License (ODbL) is a license agreement intended to allow users to freely share, modify, and use this Dataset while maintaining this same freedom for others, provided that the original source and author(s) are credited. Akihiko Kinoshita, Shohei Fujimori, Kensuke Seto, Yuki Ogura-Tsujita, Ayako Maeda, Stephan W. Gale, Junichi P. Abe, Yousuke Degawa, Tomohisa Yukawa

Supplementary material 15 Multiple sequence alignment data for rpb2 Data type fas https://binary.pensoft.net/file/1770317This dataset is made available under the Open Database License (http://opendatacommons.org/licenses/odbl/1.0/). The Open Database License (ODbL) is a license agreement intended to allow users to freely share, modify, and use this Dataset while maintaining this same freedom for others, provided that the original source and author(s) are credited. Akihiko Kinoshita, Shohei Fujimori, Kensuke Seto, Yuki Ogura-Tsujita, Ayako Maeda, Stephan W. Gale, Junichi P. Abe, Yousuke Degawa, Tomohisa Yukawa

Supplementary material 16 Supplementary references (for table S1) Data type docx https://binary.pensoft.net/file/1770318This dataset is made available under the Open Database License (http://opendatacommons.org/licenses/odbl/1.0/). The Open Database License (ODbL) is a license agreement intended to allow users to freely share, modify, and use this Dataset while maintaining this same freedom for others, provided that the original source and author(s) are credited. Akihiko Kinoshita, Shohei Fujimori, Kensuke Seto, Yuki Ogura-Tsujita, Ayako Maeda, Stephan W. Gale, Junichi P. Abe, Yousuke Degawa, Tomohisa Yukawa
