# Supplementary material for: Reflexicalyptrales ordo nov., a distinct Agaricomycetes lineage discovered from orchid mycorrhizae
Source: IMA Fungus. 2026 Sep 11;17:e201939. doi: 10.3897/imafungus.17.201939 (PMC13589298; doi:10.3897/imafungus.17.201939)
Supplement: Supplementary file 1 [file imafungus-17-e201939-s001.pdf]

Fig. S1

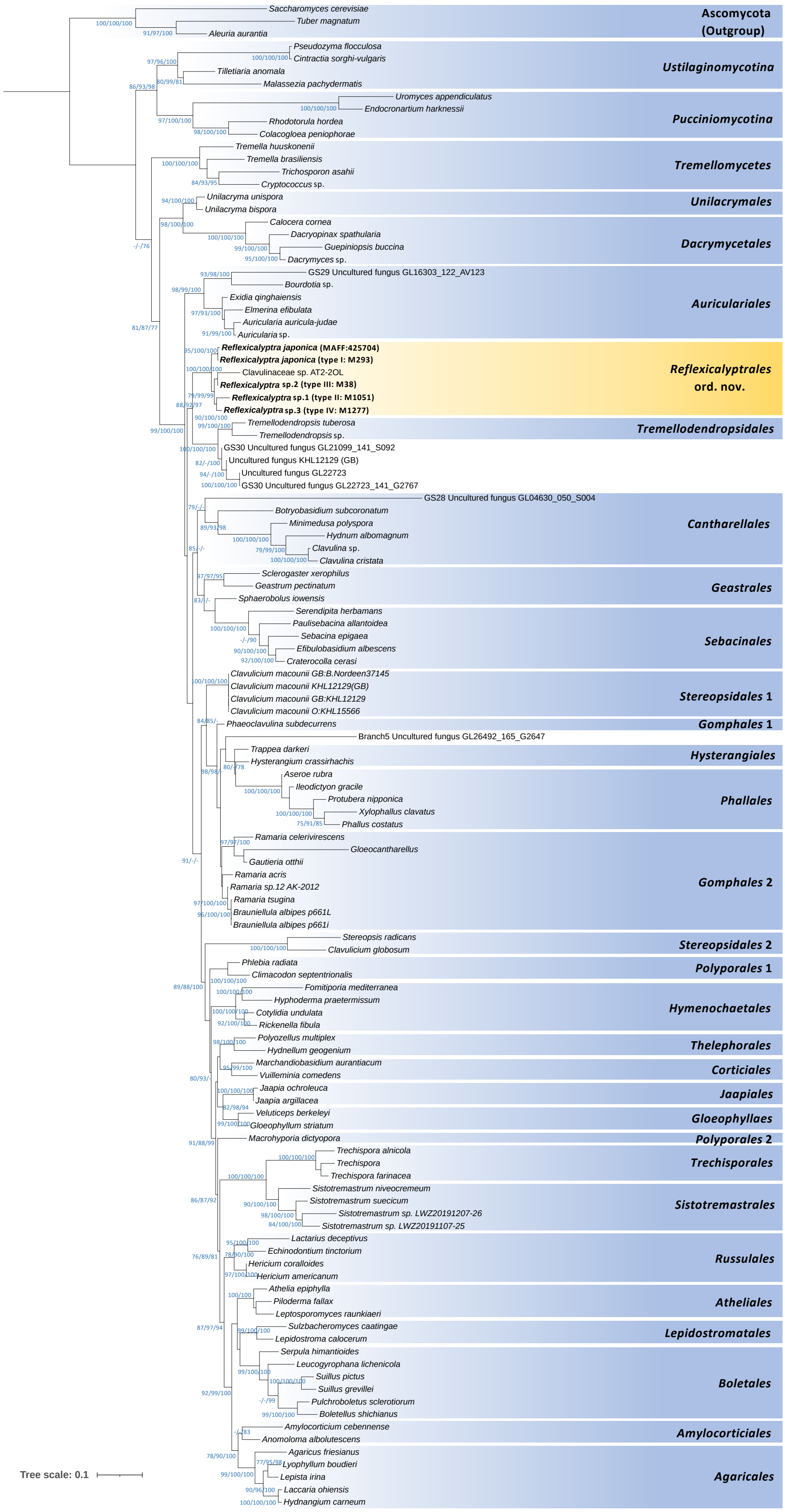

Saccharomyces cerevisiae
Tuber magnatum
Aleuria aurantia
Ascomycota (Outgroup)
Pseudozyma flocculosa
Cintractia sorghi-vulgaris
Tilletiaria anomala
Malassezia pachydermatis
Ustilaginomycotina
Uromyces appendiculatus
Endocronartium harknessii
Rhodotorula hordea
Colacogloea peniophorae
Pucciniomycotina
Tremella huuskonenii
Tremella brasiliensis
Trichosporon asahii
Cryptococcus sp.
Tremellomycetes
Unilacryma unispora
Unilacryma bispora
Unilacrymales
Calocera cornea
Dacryopinax spathularia
Guepiniopsis buccina
Dacrymyces sp.
Dacrymycetales
GS29 Uncultured fungus GL16303_122_AV123
Bourdotia sp.
Exidia qinghaiensis
Elmerina efibulata
Auricularia auricula-judae
Auricularia sp.
Auriculariales
Reflexicalyptra japonica (MAFF:425704)
Reflexicalyptra japonica (type I: M293)
Clavulinaceae sp. AT2-2OL
Reflexicalyptra sp.2 (type III: M38)
Reflexicalyptra sp.1 (type II: M1051)
Reflexicalyptra sp.3 (type IV: M1277)
Reflexicalyptrales ord. nov.
Tremellodendropsis tuberosa
Tremellodendropsis sp.
Tremellodendropsidales
GS30 Uncultured fungus GL21099_141_S092
Uncultured fungus KHL12129 (GB)
Uncultured fungus GL22723
GS30 Uncultured fungus GL22723_141_G2767
GS28 Uncultured fungus GL04630_050_S004
Botryobasidium subcoronatum
Minimedusa polyspora
Hydnum albomagnum
Clavulina sp.
Clavulina cristata
Cantharellales
Sclerogaster xerophilus
Geastrum pectinatum
Sphaerobolus iowensis
Geastrales
Serendipita herbamans
Paulisebacina allantoidea
Sebacina epigaea
Efibulobasidium albescens
Craterocolla cerasi
Sebacinales
Clavulicium macounii GB:B.Nordeen37145
Clavulicium macounii KHL12129(GB)
Clavulicium macounii GB:KHL12129
Clavulicium macounii O:KHL15566
Stereopsidales 1
Phaeoclavulina subdecurrens
Gomphales 1
Branch5 Uncultured fungus GL26492_165_G2647
Trappea darkeri
Hysterangium crassirhachis
Hysterangiales
Aseroe rubra
Ileodictyon gracile
Protubera nipponica
Xylophallus clavatus
Phallus costatus
Phallales
Ramaria celerivirescens
Gloeocantharellus
Gautieria otthii
Ramaria acris
Ramaria sp.12 AK-2012
Ramaria tsugina
Brauniellula albipes p661L
Brauniellula albipes p661i
Gomphales 2
Stereopsis radicans
Clavulicium globosum
Stereopsidales 2
Phlebia radiata
Climacodon septentrionalis
Polyporales 1
Fomitiporia mediterranea
Hyphoderma praetermissum
Cotylidia undulata
Rickenella fibula
Hymenochaetales
Polyozellus multiplex
Hydnellum geogenium
Thelephorales
Marchandiobasidium aurantiacum
Vuilleminia comedens
Corticiales
Jaapia ochroleuca
Jaapia argillacea
Jaapiales
Veluticeps berkeleyi
Gloeophyllum striatum
Gloeophyllaes
Macrohyporia dictyopora
Polyporales 2
Trechispora alnicola
Trechispora
Trechispora farinacea
Trechisporales
Sistotremastrum niveocremeum
Sistotremastrum suecicum
Sistotremastrum sp. LWZ20191207-26
Sistotremastrum sp. LWZ20191107-25
Sistotremastrales
Lactarius deceptivus
Echinodontium tinctorium
Hericium coralloides
Hericium americanum
Russulales
Athelia epiphylla
Piloderma fallax
Leptosporomyces raunkiaeri
Atheliales
Sulzbacheromyces caatingae
Lepidostroma calocerum
Lepidostromatales
Serpula himantioides
Leucogyrophana lichenicola
Suillus pictus
Suillus grevillei
Pulchroboletus sclerotiorum
Boletellus shichianus
Boletales
Amylocorticium cebennense
Anomoloma albolutescens
Amylocorticiales
Agaricus friesianus
Lyophyllum boudieri
Lepista irina
Laccaria ohiensis
Hydnangium carneum
Agaricales
Tree scale: 0.1
