# Supplementary material for: Reflexicalyptrales ordo nov., a distinct Agaricomycetes lineage discovered from orchid mycorrhizae
Source: IMA Fungus. 2026 Sep 11;17:e201939. doi: 10.3897/imafungus.17.201939 (PMC13589298; doi:10.3897/imafungus.17.201939)
Supplement: Supplementary file 2 [file imafungus-17-e201939-s002.pdf]

**Fig. S2**

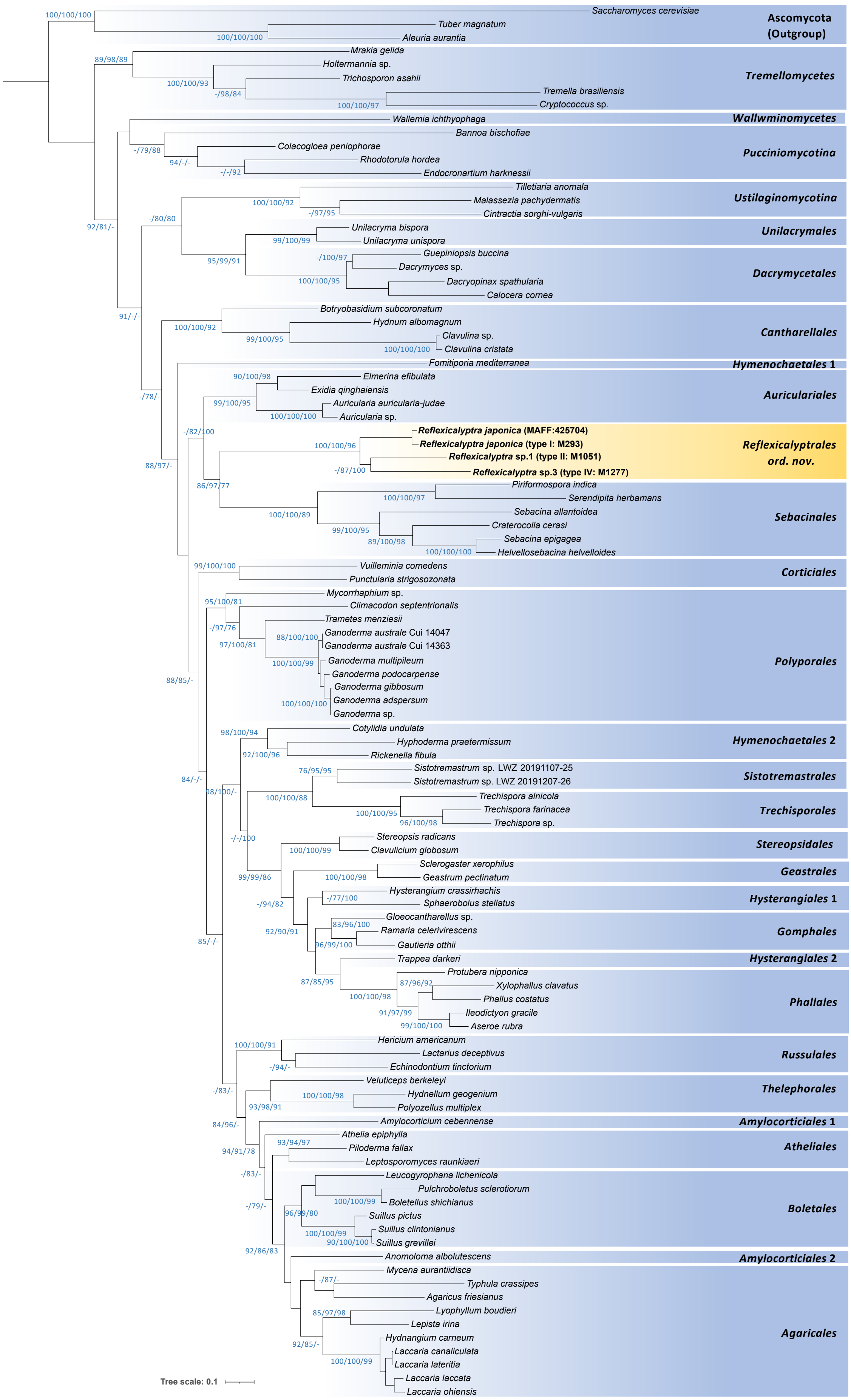
Saccharomyces cerevisiae
Tuber magnatum
Aleuria aurantia
Ascomycota (Outgroup)
Mrakia gelida
Holtermannia sp.
Trichosporon asahii
Tremella brasiliensis
Cryptococcus sp.
Tremellomycetes
Wallemia ichthyophaga
Wallwminomycetes
Bannoa bischofiae
Colacogloea peniophorae
Rhodotorula hordea
Endocronartium harknessii
Pucciniomycotina
Tilletiaria anomala
Malassezia pachydermatis
Cintractia sorghi-vulgaris
Ustilaginomycotina
Unilacryma bispora
Unilacryma unispora
Unilacrymales
Guepiniopsis buccina
Dacrymyces sp.
Dacryopinax spathularia
Calocera cornea
Dacrymycetales
Botryobasidium subcoronatum
Hydnum albomagnum
Clavulina sp.
Clavulina cristata
Cantharellales
Fomitiporia mediterranea
Hymenochaetales 1
Elmerina efibulata
Exidia qinghaiensis
Auricularia auricularia-judae
Auricularia sp.
Auriculariales
Reflexicalyptra japonica (MAFF:425704)
Reflexicalyptra japonica (type I: M293)
Reflexicalyptra sp.1 (type II: M1051)
Reflexicalyptra sp.3 (type IV: M1277)
Reflexicalyptrales ord. nov.
Piriformospora indica
Serendipita herbamans
Sebacina allantoidea
Craterocolla cerasi
Sebacina epigagea
Helvellosebacina helvelloides
Sebacinales
Vuilleminia comedens
Punctularia strigosozonata
Corticiales
Mycorrhaphium sp.
Climacodon septentrionalis
Trametes menziesii
Ganoderma australe Cui 14047
Ganoderma australe Cui 14363
Ganoderma multipileum
Ganoderma podocarpense
Ganoderma gibbosum
Ganoderma adspersum
Ganoderma sp.
Polyporales
Cotylidia undulata
Hyphoderma praetermissum
Rickenella fibula
Hymenochaetales 2
Sistotremastrum sp. LWZ 20191107-25
Sistotremastrum sp. LWZ 20191207-26
Sistotremastrales
Trechispora alnicola
Trechispora farinacea
Trechispora sp.
Trechisporales
Stereopsis radicans
Clavulicium globosum
Stereopsidales
Sclerogaster xerophilus
Geastrum pectinatum
Geastrales
Hysterangium crassirhachis
Sphaerobolus stellatus
Hysterangiales 1
Gloeocantharellus sp.
Ramaria celerivirescens
Gautieria otthii
Gomphales
Trappea darkeri
Hysterangiales 2
Protubera nipponica
Xylophallus clavatus
Phallus costatus
Ileodictyon gracile
Aseroe rubra
Phallales
Hericium americanum
Lactarius deceptivus
Echinodontium tinctorium
Russulales
Veluticeps berkeleyi
Hydnellum geogenium
Polyozellus multiplex
Thelephorales
Amylocorticium cebennense
Amylocorticiales 1
Athelia epiphylla
Piloderma fallax
Leptosporomyces raunkiaeri
Atheliales
Leucogyrophana lichenicola
Pulchroboletus sclerotiorum
Boletellus shichianus
Suillus pictus
Suillus clintonianus
Suillus grevillei
Boletales
Anomoloma albolutescens
Amylocorticiales 2
Mycena aurantiidisca
Typhula crassipes
Agaricus friesianus
Lyophyllum boudieri
Lepista irina
Hydnangium carneum
Laccaria canaliculata
Laccaria lateritia
Laccaria laccata
Laccaria ohiensis
Agaricales
Tree scale: 0.1
