# Supplementary material for: Reflexicalyptrales ordo nov., a distinct Agaricomycetes lineage discovered from orchid mycorrhizae
Source: IMA Fungus. 2026 Sep 11;17:e201939. doi: 10.3897/imafungus.17.201939 (PMC13589298; doi:10.3897/imafungus.17.201939)
Supplement: Supplementary file 3 [file imafungus-17-e201939-s003.pdf]

Fig. S3

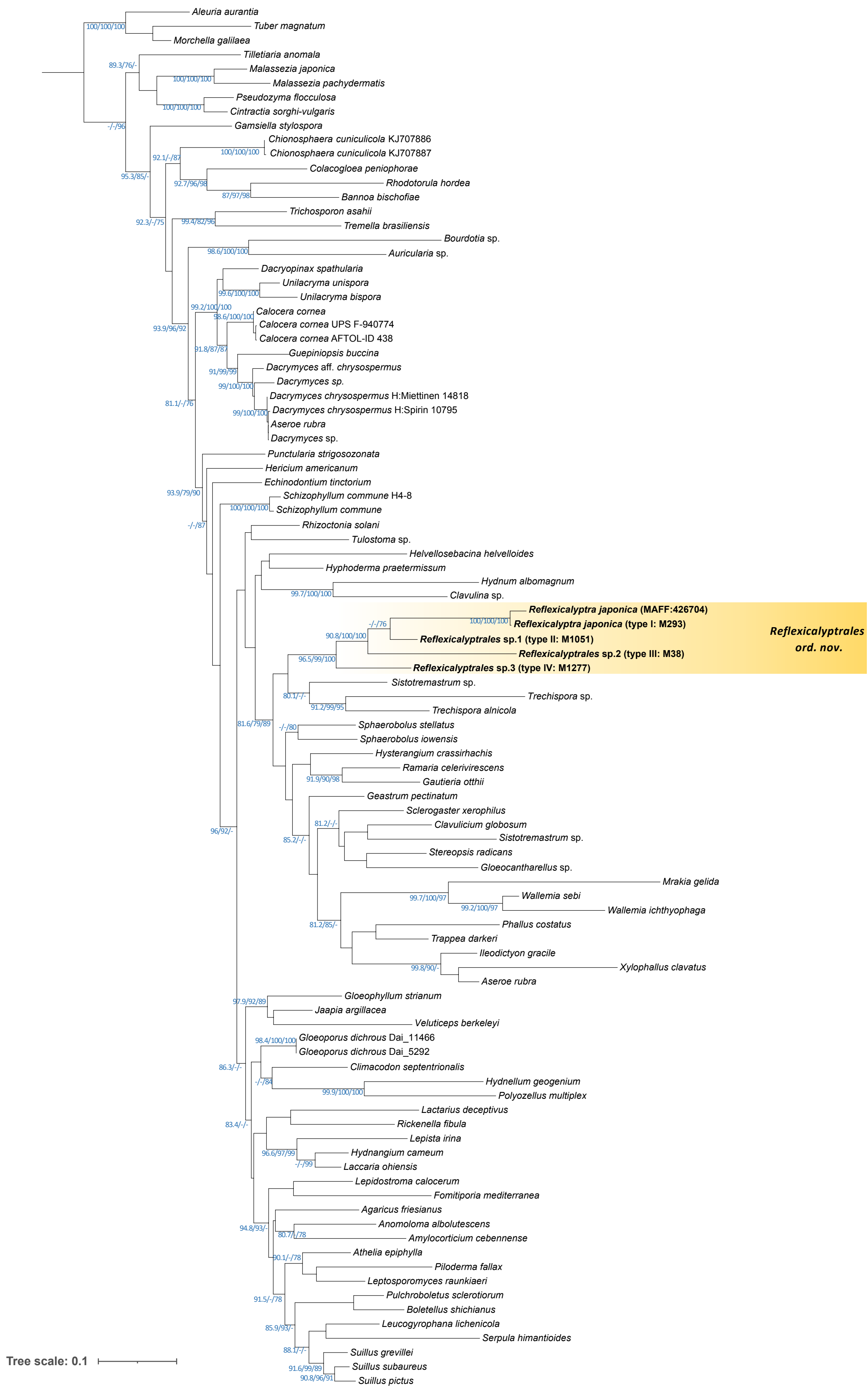
Aleuria aurantia
Tuber magnatum
Morchella galilaea
Tilletiaria anomala
Malassezia japonica
Malassezia pachydermatis
Pseudozyma flocculosa
Cintractia sorghi-vulgaris
Gamsiella stylospora
Chionosphaera cuniculicola KJ707886
Chionosphaera cuniculicola KJ707887
Colacogloea peniophorae
Rhodotorula hordea
Bannoa bischofiae
Trichosporon asahii
Tremella brasiliensis
Bourdotia sp.
Auricularia sp.
Dacryopinax spathularia
Unilacryma unispora
Unilacryma bispora
Calocera cornea
Calocera cornea UPS F-940774
Calocera cornea AFTOL-ID 438
Guepiniopsis buccina
Dacrymyces aff. chrysospermus
Dacrymyces sp.
Dacrymyces chrysospermus H:Miettinen 14818
Dacrymyces chrysospermus H:Spirin 10795
Aseroe rubra
Dacrymyces sp.
Punctularia strigosozonata
Hericium americanum
Echinodontium tinctorium
Schizophyllum commune H4-8
Schizophyllum commune
Rhizoctonia solani
Tulostoma sp.
Helvellosebacina helvelloides
Hyphoderma praetermissum
Hydnum albomagnum
Clavulina sp.
Reflexicalyptra japonica (MAFF:426704)
Reflexicalyptra japonica (type I: M293)
Reflexicalyptrales sp.1 (type II: M1051)
Reflexicalyptrales sp.2 (type III: M38)
Reflexicalyptrales sp.3 (type IV: M1277)
Reflexicalyptrales ord. nov.
Sistotremastrum sp.
Trechispora sp.
Trechispora alnicola
Sphaerobolus stellatus
Sphaerobolus iowensis
Hysterangium crassirhachis
Ramaria celerivirescens
Gautieria otthii
Geastrum pectinatum
Sclerogaster xerophilus
Clavulicium globosum
Sistotremastrum sp.
Stereopsis radicans
Gloeocantharellus sp.
Mrakia gelida
Wallemia sebi
Wallemia ichthyophaga
Phallus costatus
Trappea darkeri
Ileodictyon gracile
Xylophallus clavatus
Aseroe rubra
Gloeophyllum strianum
Jaapia argillacea
Veluticeps berkeleyi
Gloeoporus dichrous Dai_11466
Gloeoporus dichrous Dai_5292
Climacodon septentrionalis
Hydnellum geogenium
Polyozellus multiplex
Lactarius deceptivus
Rickenella fibula
Lepista irina
Hydnangium cameum
Laccaria ohiensis
Lepidostroma calocerum
Fomitiporia mediterranea
Agaricus friesianus
Anomoloma albolutescens
Amylocorticium cebennense
Athelia epiphylla
Piloderma fallax
Leptosporomyces raunkiaeri
Pulchroboletus sclerotiorum
Boletellus shichianus
Leucogyrophana lichenicola
Serpula himantioides
Suillus grevillei
Suillus subaureus
Suillus pictus
Tree scale: 0.1
