# Supplementary material for: Reflexicalyptrales ordo nov., a distinct Agaricomycetes lineage discovered from orchid mycorrhizae
Source: IMA Fungus. 2026 Sep 11;17:e201939. doi: 10.3897/imafungus.17.201939 (PMC13589298; doi:10.3897/imafungus.17.201939)
Supplement: Supplementary file 4 [file imafungus-17-e201939-s004.pdf]

Fig. S4

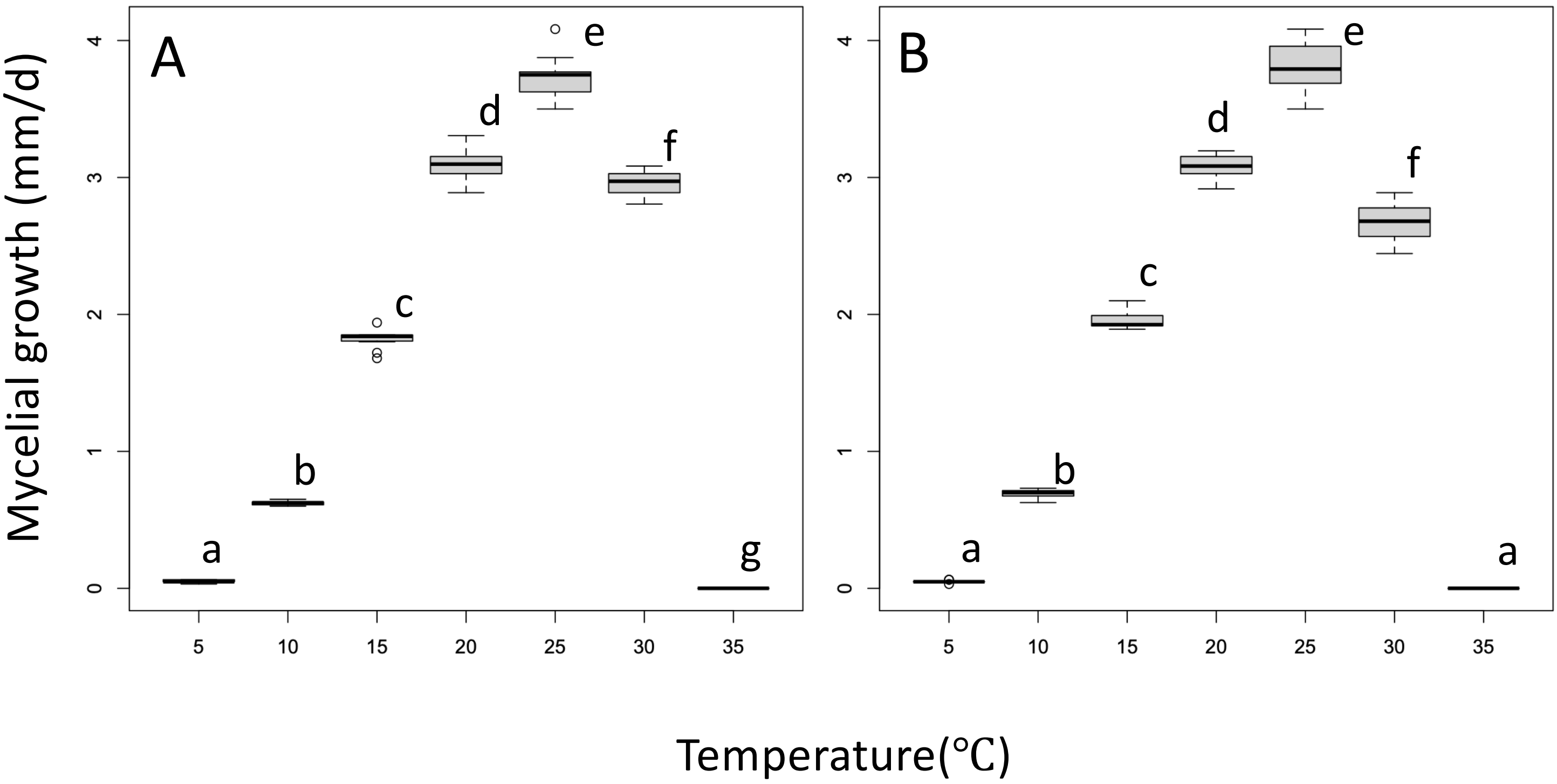
A
B
Mycelial growth (mm/d)
Temperature(℃)
0
1
2
3
4
5
10
15
20
25
30
35
a
b
c
d
e
f
g
a
b
c
d
e
f
a
