# Supplementary material for: Reflexicalyptrales ordo nov., a distinct Agaricomycetes lineage discovered from orchid mycorrhizae
Source: IMA Fungus. 2026 Sep 11;17:e201939. doi: 10.3897/imafungus.17.201939 (PMC13589298; doi:10.3897/imafungus.17.201939)
Supplement: Supplementary file 6 [file imafungus-17-e201939-s006.pdf]

Table S1. Species and Genbank accession numbers used for the phylogenetic analysis

Species	Voucher/isolate/strain	GenBank accession numbers				References
		ITS	28S	*tef1-α*	*rpb2*	
**Agaricales**						
Lepista irina	AFTOL-815	DQ221109	DQ234538	DQ028591	DQ385885	Matheny et al. (2007)
Agaricus friesianus	ZRL20151813	KX684858	KX684897	KX684919	KX684941	He et al. (2017)
Lyophyllum boudieri	BSI96/84	DQ825427	DQ825430	–	DQ825411	Hofstetter et al. (unpublished)
Laccaria ohiensis	GMM7593NZ	KU685718	KU685860	KU686121	KU686001	Wilson et al. (2017)
Hydnangium cameum	Trappe27650	KU685741	KU685892	KU686144	KU686035	Wilson et al. (2017)
**Amylocorticiales**						
Anomoloma albolutescens	CFMR: L-6088	GU187507	GU187563	GU187671	GU187768	Binder et al. (2010)
Amylocorticium cebennense	CFMR: HHB-2808	GU187505	GU187561	GU187675	GU187770	Binder et al. (2010)
**Atheliales**						
Athelia epiphylla	CFMR:FP-100564	GU187501	GU187558	GU187676	GU187771	Binder et al. (2010)
Leptosporomyces raunkiaeri	CFMR:HHB-7628	GU187528	GU187588	GU187719	GU187791	Binder et al. (2010)
Piloderma fallax	CFMR:S-12	GU187535	GU187591	GU187738	GU187797	Binder et al. (2010)
**Auriculariales**						
Auricularia sp.	AFTOL-ID 676	DQ200918	AY634277	DQ408144	DQ366278	Matheny et al. (2007)
Auricularia auricularia-judae	Dai 16353	MZ618932	MZ669900	–	MZ740047	Wu et al. (2021)
Exidia qinghaiensis	HMAS 156328	NR_172805	MW353409	–	MW358924	Wang et al. (2021)
Elmerina efibulata	Yuan 4525	MZ618945	MZ669917	–	MZ740063	Wu et al. (2021)
Bourdotia sp.	AFTOL-598	DQ200925	AY635777	DQ059760	–	Matheny et al. (2007)
**Boletales**						
Serpula himantioides	SH115	HM135653	HM135703	HM135555	HM135762	Skrede et al. (2011)
Suillus pictus	AFTOL-717	AY854069	AY684154	AY883429	AY786066	Binder et al. (unpublished)
Suillus grevillei	TDB570	KU721489	KU663262	KU721634	KU852378	Zhang et al. (2022)
Leucogyrophana lichenicola	DAOM 194172	GU187531	GU187583	GU187715	GU187789	Binder et al. (2010)
Boletellus shichianus	AFTOL-ID 532	DQ200921	AY647211	DQ408145	DQ366280	Matheny et al. (2007)
Pulchroboletus sclerotiorum	LAS-F-60333	MF098659	MF614166	MF614167	MF614169	Smith et al. (unpublished)
**Cantharellales**						
Clavulina cristata	JKU8	JN228227	JN228227	–	JN228241	Uehling et al. (2012)
Clavulina sp.	AFTOL-ID 667	DQ202266	AY745694	DQ028589	DQ366286	Matheny et al. (2007)
Hydnum albomagnum	AFTOL-ID 471	DQ218305	AY700199	DQ234568	DQ234553	Matheny et al. (2007)
Botryobasidium subcoronatum	AFTOL-ID 614	DQ200924	AY647212	–	DQ366284	Matheny et al. (2007)
Minimedusa polyspora	CBS 113.16	NR_145335	NG_057640	–	–	Kiyuna et al. (2015)
**Corticiales**						
Marchandiobasidium aurantiacum	JL391-10	HQ168397	HQ168397	–	–	Diederich et al. (2011)
Punctularia strigosozonata	AFTOL-ID 1248	DQ398958	–	DQ408147	DQ381843	Matheny et al. (2007)
Vuilleminia comedens	AFTOL-ID 1247	DQ398959	AF518666	–	DQ381844	Matheny et al. (2007)
**Dacrymycetales**						
Calocera cornea	AFTOL-ID 438	AY789083	AY701526	AY881019	AY536286	Matheny et al. (2007)
Dacrymyces sp.	AFTOL-ID 528	DQ205684	AY691892	DQ028587	DQ381845	Matheny et al. (2007)
Dacryopinax spathularia	AFTOL-ID 454	AY854070	AY701525	AY881020	AY786054	Matheny et al. (2007)
Guepiniopsis buccina	AFTOL-ID 888	DQ206986	AY745711	DQ028588	DQ385875	Matheny et al. (2007)
**Geastrales**						
Geastrum pectinatum	MEL2096557	–	DQ218602	DQ219229	DQ219051	Hosaka et al. (2006)
Sphaerobolus iowensis	SS11	AY487958	AY439014	AY487984	–	Geml et al. (2005)
Sclerogaster xerophilus	OSC49777	–	DQ218611	DQ219236	DQ219061	Hosaka et al. (2006)
Sphaerobolus stellatus	SS12	AY487959	–	DQ219237	DQ219062	Hosaka et al. (2006)
**Gloeophyllales**						
Gloeophyllum strianum	ARIZ:AN027866	HM536092	HM536063	HM536111	HM640259	Garcia-Sandoval et al. (2011)
Veluticeps berkeleyi	HHB-8594-Sp	HM536099	HM536081	HM536126	HM536125	Garcia-Sandoval et al. (2011)
**Gomphales**						
Gautieria otthii	AFTOL-ID 466	–	AF393058	AY883434	AY218486	Binder and Hibbett (2002)
Ramaria celerivirescens	SUCM-460	–	AF213074	DQ219255	DQ219077	Humpert et al. (2001)
Gloeocantharellus sp.	OSC122875	–	DQ218617	DQ219249	DQ219072	Hosaka et al. (2006)
**Hymenochaetales**						
Cotylidia undulata	IO.15.126	MT232350	MT232304	–	MT242334	Olariaga et al. (2020)
Rickenella fibula	AFTOL-ID 486	DQ241782	AY700195	DQ435794	DQ408115	Matheny et al. (2007)
Hyphoderma praetermissum	AFTOL-ID 518	AY854081	AY700185	AY885150	AY787221	Matheny and Hibbett (unpublished)
Fomitiporia mediterranea	AFTOL-ID 688	AY854080	AY684157	AY885149	AY803748	Matheny and Hibbett (unpublished)
**Hysterangiales**						
Hysterangium crassirhachis	OSC58056	DQ365632	DQ218494	DQ219153	DQ218978	Dunham et al. (2007)
Trappea darkeri	OSC65085	–	DQ218651	DQ219292	DQ219116	Dunham et al. (2007)
**Jaapiales**						
Jaapia argillacea	CBS:252.74	NR_119766	NG_042523	GU187711	GU187788	Binder et al. (2010); Schoch et al. (2014)
Jaapia ochroleuca	KHL8433	EU118637	EU118637	–	–	Larsson (2007)
**Lepidostomatales**						
Lepidostroma calocerum	Luecking 35836a	KT354923	KX431126	KF774182	KF774186	Sulzbacher et al. (2016)
Sulzbacheromyces caatingae	Sulzbacher 1479	KC170320	KC170318	–	–	Sulzbacher et al. (2012)
**Phallales**						
Aseroe rubra	OSC122632	–	DQ218625	DQ219261	DQ219082	Hosaka et al. (2006)
Phallus costatus	MB02040	–	DQ218513	DQ219279	DQ219104	Hosaka et al. (2006)
Protubera nipponica	KH-JPN11-511 (TNS)	–	KF783251	–	KF783234	Trierveiler-Pereira et al. (2014)
Xylophallus clavatus	USJ28095	KU871815	MW546308	KU871514	KU871715	Cabral et al. (unpublished)
Ileodictyon gracile		–	DQ218634	DQ219271	DQ219094	Hosaka et al. (2006)
**Polyporales**						
Climacodon septentrionalis	AFTOL-ID 767	AY854082	AY684165	AY885151	AY780941	Wang et al. (unpublished)
Phlebia radiata	FBCC1377	LN611103	LN611103	–	LN611015	Kuuskeri et al. (2015)
**Pucciniomycotina**						
Bannoa bischofiae	JCM10338	AB035721	AB082572	KJ707777	KJ708292	Hamamoto et al. (2002)
Endocronartium harknessii	AFTOL-ID 456	DQ206982	AY700193	DQ234567	DQ234551	Matheny et al. (2007)
Uromyces appendiculatus	AFTOL-ID 976	DQ411531	AY745704	DQ028595	–	Matheny et al. (2007)
Colacogloea peniophorae	AFTOL-ID 709	DQ202270	AY629313	DQ234566	DQ234550	Matheny et al. (2007)
Rhodotorula hordea	AFTOL-ID 674	DQ234557	AY631901	KJ707802	DQ234555	Matheny et al. (2007)
**Reflexicalyptales**						
Reflexicalyptra japonica	MAFF:425704	LC820841	LC820842	LC820843	LC820844	This study
Reflexicalyptra japonica	M293 (type I)	AB712269	AB712269	LC820845	LC820846	Nomura et al. (2010); this study
Reflexicalyptrales sp.1	M1051 (type II)	AB712270	AB712270	LC820847	LC820848	Nomura et al. (2010); this study
Reflexicalyptrales sp.2	M38 (type III)	AB712271	AB712271	LC820849	–	Nomura et al. (2010); this study
Reflexicalyptrales sp.3	M1277 (type IV)	AB712272	AB712272	LC820850	LC820851	Nomura et al. (2010); this study
**Russulales**						
Hericium coralloides	NH282	AF506459	AF506459	–	–	Larsson and Larsson (2003)

Hericium americanum	AFTOL-ID 469	DQ206987	DQ411538	DQ028585	DQ408127	Matheny et al. (2007)
Echinodontium tinctorium	AFTOL-ID 455	AY854088	AF393056	AY885157	AY218482	Matheny and Hibbett (unpublished)
Lactarius deceptivus	AFTOL-ID 682	AY854089	AY631899	AY885158	AY803749	Matheny and Hibbett (unpublished)
**Sebacinales**						
Craterocolla cerasi	TUB 020203	KF061265	KF061265	–	KF061300	Oberwinkler et al. (2014)
Helvellosebacina helvelloides	TU115570	–	–	KF313910	KF313878	Tedersoo et al. (2014)
Efibulobasidium albescens	RJB 12952	AF384860	AF384860	–	–	Lim and Berbee (unpublished)
Sebacina allantoidea	RoKi 179	KF061266	KF061266	–	KF061301	Oberwinkler et al. (2014)
Sebacina epigagea	TUB 019689	JQ665467	JQ665467	–	JQ665571	Riess et al. (2013)
Serendipita herbamans	S1	KF061285	KF061285	–	KF061317	Riess et al. (2014)
Piriformospora indica	AFTOL-ID 612	DQ411527	–	–	DQ408131	Nilsson et al. (unpublished)
**Sistotremastrales**						
Sistotremastrum niveocremeum	Larsson 13727	MN937563	MN937563	–	–	Spirin et al. (2021)
Sistotremastrum suecicum	KHL-11849	MN937571	MN937571	–	–	Spirin et al. (2021)
Sistotremastrum sp.	LWZ 20191107-25	MW477771	MW474864	MW478703	MW478712	Liu et al. (2021)
Sistotremastrum sp.	LWZ 20191207-26	OM523377	OM339205	OM416796	OM416816	Liu et al. (2021)
**Stereopsidales**						
Clavulicium globosum	GB:KHL12592	KC203495	KC203495	KC203515	KC203501	Sjökvist et al. (2014)
Stereopsis radicans	GB:699Ryvarden	KC203488	KC203488	KC203508	KC203502	Sjökvist et al. (2014)
**Thelephorales**						
Polyozellus multiplex	AFTOL-ID 677	DQ411528	AY634275	DQ028596	DQ408134	Matheny et al. (unpublished)
Hydnellum geogenium	AFTOL-ID 680	DQ218304	AY631900	DQ059053	DQ408133	Matheny et al. (2007)
**Trechisporales**						
Trechispora alnicola	AFTOL-ID 665	DQ411529	AY635768	DQ059052	DQ408135	Matheny and Hibbett (unpublished)
Trechispora sp.	AFTOL-ID 678	DQ411534	AY647217	DQ059051	DQ408136	Matheny and Hibbett (unpublished)
Trechispora farinacea	TUB 011825	EU909231	EU909231	–	KF211261	Krause et al. (2011)
**Tremellodendropsidales**						
Tremellodendropsis tuberosa	UBC:F31377	KU900861	KU900861	–	–	Berbee et al. (2016)
Tremellodendropsis sp.	MES-1670	KY462517	KY487063	–	–	Truong et al. (2017)
**Unilacrymales**						
Unilacryma unispora	UPS F-941298	MN595669	MN595669	MN580366	MN580311	Zamora and Ekman (2020)
Unilacryma bispora	UPS F-941254	MN595670	MN595670	MN580367	MN580312	Zamora and Ekman (2020)
**Tremellales**						
Cryptococcus sp.	AFTOL-ID 719	DQ205681	AY646103	–	DQ408137	Matheny et al. (2007)
Holtermannia sp.	21825S-11	OP470257	OP470161	–	OP771444	Wang and Jiang (unpublished)
Mrakia gelida	CBS5272	NR_163504	AF189831	KF037187	KF036926	Diaz and Fell (2000)
Tremella huuskonenii	HL12Sb	KR857043	KR857075	–	–	Lindgren et al. (2015)
Tremella brasiliensis	CBS 6966	NR_119454	NG_042360	KF037200	KF036938	Schoch et al. (2014)
Trichosporon asahii	CBS2497	KY105711	KY109923	KF037216	KF036955	Vu et al. (2016)
**Ustilaginomycetes**						
Cintractia sorghi-vulgaris	AFTOL-ID 867	DQ200931	AY745726	DQ028590	DQ234549	Matheny et al. (2007)
Pseudozyma flocculosa	AFTOL-ID 864	DQ411535	AY745712	DQ028598	DQ408142	Matheny et al. (2007)
Tilletiaria anomala	AFTOL-ID 865	DQ234558	AY745715	DQ835991	AY803750	Matheny et al. (2007)
Malassezia pachydermatis	AFTOL-ID 856	DQ411532	AY745724	DQ028594	DQ408140	Matheny et al. (unpublished)
**Wallemiomycestes**						
Wallemia ichthyophaga	AFTOL-ID1901	–	DQ847516	DQ847525	DQ847519	Matheny et al. (2007)
Wallemia sebi	AFTOL-ID1910	–	DQ847518	DQ847527	DQ847524	Matheny et al. (2007)
**Ascomycota**						
Aleuria aurantia	AFTOL-65	DQ491495	AY544654	DQ466085	DQ247785	Matheny et al. (2007)
Morchella galilaea	M308	JQ723083	GU551210	GU551154	GU551322	Du et al. (2012)
Tuber magnatum	GB13	JQ925646	JQ925701	JX022592	JQ954513	Bonito et al. (2013)
Saccharomyces cerevisiae	S288c	–	AH005487	NM_001184177	NM_001183570	Bayev et al. 1980); Engel et al. (2022)

Notes: References cited in this table are listed in the Supplementary References.
