# Supplementary material for: Reflexicalyptrales ordo nov., a distinct Agaricomycetes lineage discovered from orchid mycorrhizae
Source: IMA Fungus. 2026 Sep 11;17:e201939. doi: 10.3897/imafungus.17.201939 (PMC13589298; doi:10.3897/imafungus.17.201939)
Supplement: Supplementary file 7 [file imafungus-17-e201939-s007.pdf]

Table S2. Top five BLAST hit sequences of 28S, *rpb2* and *tef1-α* sequences of four types uAX (accessed 14 Oct 2025)

Species	Order	Voucher/isolate/strain	Genbank accession number	Sequence similarities (%)					E-value				
				MAFF:425 704	M293 (type I)	M1051 (type II)	M38 (type III)	M1277 (type IV)	MAFF:425 704	M293 (type I)	M1051 (type II)	M38 (type III)	M1277 (type IV)
LSU													
Brauniellula albipes	Boletales	p661i	EU669381.1	93.0					0.0				
Brauniellula albipes	Boletales	p661L	EU669423.1	93.0					0.0				
Clavulinaceae sp.	Cantharellales	AT2-2OL	AB831869.1	97.0	93.1	93.1	97.3	96.1	0.0	0.0	0.0	0.0	0.0
Phaeoclavulina subdecurrens	Cantharellales	AMB 18548	MT053217.1	93.0					0.0				
Ramaria tsugina	Gompales	WTU-F-043052	KX671004.1	93.0					0.0				
Ramaria acris	Gomphales	UT-36052-T	OM238175.1			91.9					0.0		
Clavulicium macounii	Stereopsidales	O:KHL15566	KC203500.1		93.1	91.9	93.2	92.8		0.0	0.0	0.0	0.0
Clavulicium macounii	Stereopsidales	AK-2012	JQ408244.1		93.1		93.2			0.0		0.0	
Clavulicium macounii	Stereopsidales	GB:B.Nordeen37145	MT537069.1		93.1		93.2			0.0		0.0	
Clavulicium macounii	Stereopsidales	KHL12129 (GB)	JX316518.1		93.1	92.3	93.2			0.0	0.0	0.0	
Clavulicium macounii	Stereopsidales	GB:KHL12129	KC203494.1					92.7					0.0
Clavulicium macounii	Stereopsidales	GB:B.Nordeen37145	KC203492.1					92.7					0.0
Clavulicium macounii	Stereopsidales	KHL12129 (GB)	JN649333.1					92.6					0.0
Uncultured fungus	Unknown	GL22723	KY687730.1			91.9					0.0		
*rpb*2													
Hydnangium carneum	Agaricales	Trappe27650	KU686035.1			79.3					8.00E-92		
Laccaria laccata	Agaricales	GMM7586	KU686000.1			80.8					2.00E-92		
Laccaria lateritia	Agaricales	GMM7250	KU685954.1			80.1					3.00E-91		
Laccaria ohiensis	Agaricales	GMM7593NZ	KU686001.1			80.6					3.00E-91		
Laccaria canaliculata	Agaricales	GMM7267	KU685960.1			80.1					6.00E-101		
Mycena aurantiidisca	Agaricales	AFTOL-ID 16	DQ474122.1	79.5					3.00E-99				
Boletellus shichianus	Boletales	AFTOL-ID 532	DQ366280.1					77.2					1.00E-83
Ganoderma podocarpense	Polyporales	JV 1504/126	MZ345737.1	77.6					8.00E-90				
Ganoderma australe	Polyporales	Cui 14363	MH115395.1	77.5					8.00E-90				
Ganoderma australe	Polyporales	Cui 14047	MH115394.1	77.5					8.00E-90				
Leucogyrophana lichenicola	Boletales	DAOM 194172	GU187789.1					77.7					1.00E-87
Suillus clintonianus	Boletales	HHB-6988-Sp	XM_041351227.1					77.0					1.00E-108
Suillus grevillei	Boletales	TDB570	KU852378.1					77.3					1.00E-87
Ganoderma sp.	Polyporales	YFS-2021h voucher Cui 14454	MZ245408.1		78.1					5.00E-97			
Ganoderma adspersum	Polyporales	YFS-2021h voucher Cui 14453	MZ245407.1		78.1					2.00E-95			
Ganoderma gibbosum	Polyporales	isolate 42	MK340899.1		78.0					4.00E-93			
Ganoderma multipileum	Polyporales	LGAM 336 - ACAM DD2118	MG837806.1		77.8					6.00E-93			
Trametes menziesii	Polyporales	Cui 16808	OK665335.1					78.8					6.00E-91
Typhula crassipes	Polyporales	IO.14.83	MT242342.1		79.0					5.00E-97			
Mycorrhaphium sp.	Polyporales	MCW:429/13	MH475313.1	78.6					5.00E-92				
*tef*1-α													
Schizophyllum commune	Agaricales	-	LR213609.1			86.1					1.00E-140		
Schizophyllum commune	Agaricales	H4-8	XM_003037215.2			86.3					1.00E-140		
Tulostoma sp.	Agaricales	Finy12	KU843902.1	77.5					1.00E-75				
Chionosphaera cuniculicola	Agaricostilbales	CBS 10065	KJ707887	77.0					2.00E-74				
Chionosphaera cuniculicola	Agaricostilbales	CBS 10063	KJ707886.1	77.0					2.00E-74				
Suillus subaureus	Boletales	MN1	XM_041329927.1			86.7					4.00E-144		
Rhizoctonia solani	Cantharellales	AG-1 IA	XM_043320671.1			86.3	79.1				4.00E-144	1.00E-85	
Rhizophlyctis sp.	Cantharellales	C-10	AB077105.1				78.8					5.00E-79	
Calocera cornea	Dacrymycetales	CWU6922	MW130299.1		82.0					8.00E-107			
Calocera cornea	Dacrymycetales	UPS F-940774	MN580325.1		81.9					2.00E-103			
Dacrymyces chrysospermus	Dacrymycetales	H:Miettinen 14818	MW130305.1					87.5					2.00E-147
Dacrymyces aff. *chrysospermus*	Dacrymycetales	UPS F-593536	MN580330.1					87.2					2.00E-142
Dacrymyces sp.	Dacrymycetales	FPL8953	DQ028587.1					87.3					4.00E-149
Dacrymyces sp.	Dacrymycetales	Da9pm1	ON661052.1		82.2			86.4		2.00E-107			1.00E-140
Dacrymyces sp.	Dacrymycetales	FPL8953	DQ028587.1		82.1					2.00E-107			
Dacrymyces chrysospermus	Dacrymycetales	H:Spirin 10795	MW130306.1		81.5			87.4		2.00E-102			4.00E-149
Malassezia japonica	Malasseziales	CBS 9431	XM_060265987.1				77.6					2.00E-72	
Gamsiella stylospora	Mortierellales	NRRL_28272	MN878268.1			86.9					2.00E-147		
Hesseltinella vesiculosa	Mucorales	-	AF157255.1				79.0					5.00E-84	
Gloeoporus dichrous	Polyporales	Dai_5292	OP352400.1	83.0					3.00E-72				
Gloeoporus dichrous	Polyporales	Dai_11466	OP352399.1	83.0					3.00E-72				
