# Supplementary material for: Reflexicalyptrales ordo nov., a distinct Agaricomycetes lineage discovered from orchid mycorrhizae
Source: IMA Fungus. 2026 Sep 11;17:e201939. doi: 10.3897/imafungus.17.201939 (PMC13589298; doi:10.3897/imafungus.17.201939)
Supplement: Supplementary file 8 [file imafungus-17-e201939-s008.pdf]

Table S3. Accession and isolate numbers of four novel lineages identified by Tedersoo et al. (2017), which were added to the 28 rDNA phylogenetic analysis

Lineage name	isolate	NCBI accession number
GS30	GL21099_141_S092	KY687729
GS30	GL22723_141_S126	KY687730
GS30	GL22723_141_G2767	KY687731
GS29	GL16303_122_AV123	KY687703
branch5	GL26492_165_G2647	KY687759
GS28	GL04630_050_S004	KY687594
