# Supplementary material for: Reflexicalyptrales ordo nov., a distinct Agaricomycetes lineage discovered from orchid mycorrhizae
Source: IMA Fungus. 2026 Sep 11;17:e201939. doi: 10.3897/imafungus.17.201939 (PMC13589298; doi:10.3897/imafungus.17.201939)
Supplement: Supplementary file 9 [file imafungus-17-e201939-s009.pdf]

Table S4. The number of OTUs, sequence length, conserved, parsimony informative and variable sites used for phylogenetic analysis

Dataset	Number of OTUs	Sequence length[1]	The number of sites			Best-fit model[2]
			Conserved	Parsimony informative	Variable	
4 loci						
28S	93	530 (982)	188	296	371	TIM3+I+G4
5.8s	83	159 (209)	72	57	90	TIM3u+G4
rpb2	70	368 (4633)	117	198	289	TIM3+F+I+G4
tef1-α	78	485 (2885)	50	403	463	GTR+I+G4
Individual loci						
28S	121	951	354	485	706	GTR+F+I+G4
ITS	82	743	500	191	350	TIM3e+I+G4
tef1-α						GTR+I+G[3]
1st codon	100	344	191	123	236	GTR+F+R4
2nd codon	100	344	232	82	215	TVM+F+I+R3
3rd codon	100	344	10	329	342	GTR+F+I+R5
rpb2						GTR+I+G[3]
1st codon	113	354	86	232	329	GTR+F+I+G4
2nd codon	113	353	134	160	289	GTR+F+R4
3rd codon	113	353	6	347	353	TPM2u+F+I+R5

[1] The sequence length before Gblocks is indicated in parentheses

[2] The best-fit model is determined according to the Bayesian Information Criterion (BIC) by ModelFinder

[3] The best-fit model for the locus, determined based on BIC
