# Supplementary material for: Reflexicalyptrales ordo nov., a distinct Agaricomycetes lineage discovered from orchid mycorrhizae
Source: IMA Fungus. 2026 Sep 11;17:e201939. doi: 10.3897/imafungus.17.201939 (PMC13589298; doi:10.3897/imafungus.17.201939)
Supplement: Supplementary file 10 [file imafungus-17-e201939-s010.pdf]

Table S5. BLAST results of the ITS rDNA for four types of uAX against UNITE, INSD, and HTS sequences (accessed 15 Oct 2025)

Query	Representative sequences	SH code	Country	Origin	Latitude, longitude (N, E)	Altitude (m)	Putative taxa identified by UNITE	Score	Similarity (%)	E-value
M293 (type I)	AB712269	SH0840430.10FU	Japan: Tokushima, Kochi, Miyazaki, Kagoshima	*Nervilia nipponica* mycorrhiza			Agaricomycetes	1115.8	100	0
	AB712270	SH0840429.10FU	Japan: Tokyo	*Nervilia nipponica* mycorrhiza			Agaricomycetes	770.7	89.06	0
	AB712271	SH0840451.10FU	Japan: Miyzaki	*Nervilia nipponica* mycorrhiza			Agaricomycetes	698.6	87.05	0
	DQ294946	SH0840445.10FU	Zimbabwe	*Zea mays* roots	–	–	*Sparassis*	779.3	92.87	0
	JQ247540	SH0840448.10FU	United States	Soil DNA	38.938196, -76.552720		Basidiomycota	710.6	97.32	0
	KT581823	SH0840444.10FU	Mexico	*Quercus deserticola* litter	19.538400, -101.299000	2288	Agaricomycetes	947.5	96.58	0
	KT581937	SH0840444.10FU	Mexico	Soil DNA	19.32600, -101.18360	–	Agaricomycetes	947.5	96.58	0
	KU195550	SH0840438.10FU	Tanzania	*Allanblackia stuhlmannii* roots	-5.496000, 36.077000	1278	Fungi	873.7	94.56	0
	KU195593	SH0840453.10FU	Tanzania	*Allanblackia stuhlmannii* roots	-5.76900000, 37.74400000	–	Agaricomycetes	865.1	94.24	0
	MH005872	SH0840433.10FU	China	Terestrial orchid mycorrhizal root	–	–	Agaricomycetes	841.1	93.17	0
	UDB05156310	SH0840444.10FU	Colombia	Soil DNA	11.28425399, -74.011557	152	Polyporales	798.2	96.18	0
	UDB05156312	SH0840444.10FU	Buritaca	Soil DNA	11.28425399, -74.011557	152	Polyporales	793	95.99	0
	UDB05157662	SH0840456.10FU	Cuba	Soil DNA	20.1954, -76.4524	300	Polyporales	714	93.42	0
	UDB05157928	SH0840447.10FU	India	Soil DNA	25.5024, 91.2845	1480	Polyporales	753.5	94.8	0
	UDB05158298	SH0840447.10FU	India	Soil DNA	26.6433, 90.2611	120	Polyporales	753.5	94.8	0
	UDB05158608	SH0840444.10FU	Brazil	Soil DNA	-3.9847, -38.6251	135	Polyporales	786.1	95.63	0
	UDB05158614	SH0840444.10FU	Brazil	Soil DNA	-4.4099,-38.9113	200	Polyporales	782.7	95.61	0
	UDB05159833	SH0840444.10FU	Mexico	Soil DNA	18.581583, -95.078767	320	Polyporales	798.2	96.18	0
	UDB05160216	SH0840457.10FU	Papua New Guinea	Soil DNA	-5.010067, 145.768966	81	Polyporales	719.2	93.31	0
	UDB07353583	SH0840446.10FU	Brazil	Soil DNA	-12.8, -39.2	150	*Sparassis*	698.6	92.84	0
	UDB07353597	SH0840449.10FU	Benin	Soil DNA	11.62705, 3.17812	207	*Sparassis*	703.7	93.04	0
	UDB07353607	SH0840440.10FU	Réunion	Soil DNA	-21.36314, 55.75824	12	*Sparassis*	757	95.15	0
	UDB07353608	SH0840440.10FU	Réunion	Soil DNA	-21.36314, 55.75824	12	*Sparassis*	751.8	94.95	0
	UDB07353620	SH0840453.10FU	Cameroon	Soil DNA	5.2, 10.0	718	*Sparassis*	750.1	94.94	0
	UDB07353624	SH0840433.10FU	Thailand	Soil DNA	19.88388889, 100.09361111	440	*Sparassis*	696.9	92.23	0
	UDB07353654	SH0840433.10FU	Thailand	Soil DNA	19.88444444, 100.0925	440	*Sparassis*	696.9	92.23	0
	UDB07353656	SH0840433.10FU	Thailand	Soil DNA	19.88444444, 100.0925	440	*Sparassis*	700.3	92.41	0
	UDB07353658	SH0840433.10FU	Thailand	Soil DNA	19.88444444, 100.0925	440	*Sparassis*	702	92.42	0
	UDB07353663	SH0840433.10FU	Thailand	Soil DNA	19.88444444, 100.0925	440	*Sparassis*	698.6	92.25	0
	UDB07353772	SH0840436.10FU	Tanzania	Soil DNA	-3.3622, 37.3587	805	*Sparassis*	712.3	93.26	0
M1051 (type II)	AB712269	SH0840430.10FU	Japan: Tokushima, Kochi, Miyazaki, Kagoshima	*Nervilia nipponica* mycorrhiza			Agaricomycetes	770.7	89.06	0
	AB712270	SH0840429.10FU	Japan: Tokyo	*Nervilia nipponica* mycorrhiza			Agaricomycetes	1124.3	100	0
	AB712271	SH0840451.10FU	Japan: Miyzaki	*Nervilia nipponica* mycorrhiza			Agaricomycetes	691.7	86.7	0
	AB831854	SH0840458.10FU	Japan: Tochigi	*Neottia makinoana* mycorrhiza	36.707446, 139.703838		Corticiaceae	681.4	86.31	0
	DQ294946	SH0840445.10FU	Zimbabwe	*Zea mays* roots	–	–	*Sparassis*	645.4	88.01	0
	GQ219847	SH0957648.10FU	Germany	Soil DNA	51.21667000, 10.45000000	–	Trechisporales	671.1	86.1	0
	KJ188622	SH0957648.10FU	West Bohemia	*Neottia ovata* mycorrhizal seedling (protocorm)	49.0329, 13.4951	–	Agaricomycetes	604.2	85.33	0
	KT581823	SH0840444.10FU	Mexico	*Quercus deserticola* litter	19.538400, -101.299000	2288	Agaricomycetes	722.6	89	0
	KT581937	SH0840444.10FU	Mexico	Soil DNA	19.32600, -101.18360	–	Agaricomycetes	722.6	89	0
	KU195550	SH0840438.10FU	Tanzania	*Allanblackia stuhlmannii* roots	-5.496, 36.077	1278	Fungi	710.6	89.09	0
	KU195593	SH0840453.10FU	Tanzania	*Allanblackia stuhlmannii* roots	-5.76900000, 37.74400000	–	Agaricomycetes	726	89.58	0
	LC028214	SH0840429.10FU	Japan	*Cerasus sargentii* roots	–	–	Fungi	720.9	99.76	0
	UDB0193054	SH0840442.10FU	Estonia	Soil DNA	58.41593156, 25.23269238	29	Eukaryota	703.7	92.59	0
	UDB05156310	SH0840444.10FU	Colombia	Soil DNA	11.28425399, -74.011557	152	Polyporales	587	88.87	0
	UDB05156312	SH0840444.10FU	Buritaca	Soil DNA	11.28425399, -74.011557	152	Polyporales	581.8	88.67	0
	UDB05157662	SH0840456.10FU	Cuba	Soil DNA	20.1954, -76.4524	300	Polyporales	564.7	88.06	0
	UDB05158608	SH0840444.10FU	Brazil	Soil DNA	-3.9847, -38.6251	135	Polyporales	585.3	88.72	0
	UDB05158614	SH0840444.10FU	Brazil	Soil DNA	-4.4099, -38.9113	200	Polyporales	583.6	88.69	0
	UDB05159833	SH0840444.10FU	Mexico	Soil DNA	18.581583, -95.078767	320	Polyporales	593.9	89.08	0
	UDB05160388	SH0840441.10FU	South Africa	Soil DNA	-33.97862, 22.61098	48	Polyporales	729.5	93.54	0
	UDB07353597	SH0840449.10FU	Benin	Soil DNA	11.62705, 3.17812	207	*Sparassis*	569.8	88.51	0
	UDB07353607	SH0840440.10FU	Réunion	Soil DNA	-21.36314, 55.75824	12	*Sparassis*	585.3	88.85	0
	UDB07353608	SH0840440.10FU	Réunion	Soil DNA	-21.36314, 55.75824	12	*Sparassis*	580.1	88.65	0
	UDB07353618	SH0840434.10FU	Costa Rica	Soil DNA	8.64198, -83.72496	55	*Sparassis*	566.4	87.84	0
	UDB07353620	SH0840453.10FU	Cameroon	Soil DNA	5.2, 10.0	718	*Sparassis*	602.4	89.59	0
	UDB07353626	SH0840433.10FU	Thailand	Soil DNA	19.88388889, 100.09361111	440	*Sparassis*	566.4	87.84	0
	UDB07353650	SH0840455.10FU	Thailand	Soil DNA	19.88444444, 100.0925	440	*Sparassis*	568.1	88.49	0
	UDB07353652	SH0840455.10FU	Thailand	Soil DNA	19.88444444, 100.0925	440	*Sparassis*	571.5	88.67	0
	UDB07353658	SH0840433.10FU	Thailand	Soil DNA	19.88444444, 100.0925	440	*Sparassis*	573.3	88.18	0
	UDB07353772	SH0840436.10FU	Tanzania	Soil DNA	-3.3622, 37.3587	805	*Sparassis*	568.1	88.24	0
M38 (type III)	AB712269	SH0840430.10FU	Japan: Tokushima, Kochi, Miyazaki, Kagoshima	*Nervilia nipponica* mycorrhiza			Agaricomycetes	698.6	87.05	0
	AB712271	SH0840451.10FU	Japan: Miyzaki	*Nervilia nipponica* mycorrhiza			Agaricomycetes	1114	100	0
	AB831854	SH0840458.10FU	Japan: Tochigi	*Nervilia nipponica* mycorrhiza			Corticiaceae	1033.4	97.39	0
	GQ219847	SH0957648.10FU	Germany	Soil DNA	51.21667000, 10.45000000		Trechisporales	700.3	87.07	0
	KJ188615	SH0957641.10FU	Czechia	*Neottia ovata* mycorrhiza	49.0917, 13.1958	–	Trechisporales	593.9	84.92	0
	KJ188622	SH0957648.10FU	West Bohemia	*Neottia ovata* mycorrhizal seedling (protocorm)	49.0329, 13.4951	–	Agaricomycetes	633.3	86.36	0
	KT581823	SH0840444.10FU	Mexico	*Quercus deserticola* litter	19.538400, -101.299000	2288	Agaricomycetes	645.4	86.7	0
	KT581937	SH0840444.10FU	Mexico	Soil DNA	19.32600, -101.18360	–	Agaricomycetes	645.4	86.7	0
	KU195550	SH0840438.10FU	Tanzania	*Allanblackia stuhlmannii* roots	-5.496, 36.077	1278	Fungi	647.1	87.11	0
	KU195593	SH0840453.10FU	Tanzania	*Allanblackia stuhlmannii* roots	-5.76900000, 37.74400000	–	Agaricomycetes	657.4	87.44	0
	KY574436	SH0957647.10FU	Mexico	*Quercus* ectomycorrhiza	19.981289, -101.197880	–	Agaricomycetes	678	86.54	0
	UDB0187175	SH0957638.10FU	Estonia	Soil DNA	58.63443, 23.43938	4	Corticiales	599	89.02	0
	UDB026606	SH0957642.10FU	Morocco	*Cistus creticus* ectomycorrhiza	33.77287002 , -4.98149104	1293	Agaricomycetes	592.1	85.25	0
	UDB0409288	SH0957638.10FU	Estonia	Soil DNA	58.46726, 25.44357	78	Corticiales	590.4	88.65	0
	UDB0523134	SH0957638.10FU	Estonia	Soil DNA	58.01829, 22.17463	58	Eukaryota	600.7	88.91	0
	UDB0588538	SH0957638.10FU	Estonia	Soil DNA	59.06406, 24.67274	86	Corticiales	590.4	88.65	0
	UDB0613907	SH0957638.10FU	Estonia	Soil DNA	58.67427, 24.61056	42	Corticiales	590.4	88.65	0
	UDB06367314	SH0957638.10FU	Morocco	Soil DNA	31.68288, -7.03608	1412	Trechisporales	593.9	88.82	0
	UDB06368216	SH0957638.10FU	Estonia	Soil DNA	59.06406, 24.67274	86	Trechisporales	590.4	88.65	0
	UDB06368740	SH0957638.10FU	Estonia	Soil DNA	58.62853367, 23.4343709	17	Trechisporales	590.4	88.65	0
	UDB06369030	SH0957638.10FU	Estonia	Soil DNA	59.47013283, 25.27921329	40	Trechisporales	595.6	88.85	0
	UDB06369711	SH0957638.10FU	Estonia	Soil DNA	58.63443, 23.43938	4	Trechisporales	599	89.02	0
	UDB06377016	SH0957650.10FU	Guatemala	Soil DNA	14.5222, -90.8779	3050	Trechisporales	629.9	90.87	0
	UDB06385573	SH0957638.10FU	Italy	Soil DNA	46.40722, 11.49639	1211	Trechisporales	599	89.02	0
	UDB06385576	SH0957638.10FU	Italy	Soil DNA	46.40722, 11.49639	1211	Trechisporales	590.4	88.65	0
	UDB06387869	SH0957638.10FU	Estonia	Soil DNA	59.10435, 24.34035	47	Trechisporales	590.4	88.65	0
	UDB06387870	SH0957638.10FU	Estonia	Soil DNA	59.10435, 24.34035	47	Trechisporales	599	89.02	0
	UDB06388194	SH0957643.10FU	Estonia	Soil DNA	59.10435, 24.34035	47	Trechisporales	636.8	90.66	0
	UDB06388747	SH0840431.10FU	Tanzania, United Republic	Soil DNA	-3.3622, 37.3587	805	Trechisporales	641.9	90.58	0
	UDB06389605	SH0957639.10FU	Mexico	Soil DNA	16.15137, -90.89482	177	Trechisporales	631.6	90.33	0
M1277 (type IV)	AB712271	SH0840451.10FU	Japan	*Nervilia nipponica* mycorrhiza			Agaricomycetes	679.7	86.03	0
	AB712272	SH0957645.10FU	Japan	*Nervilia nipponica* mycorrhiza			Agaricomycetes	1157	100	0
	AB831854	SH0840458.10FU	Japan	*Nervilia nipponica* mycorrhiza	36.707446, 139.703838		Corticiaceae	669.4	85.74	0
	GQ219847	SH0957648.10FU	Germany	Soil DNA	51.21667000, 10.45000000	–	Trechisporales	837.6	90.68	0
	GQ921808	SH0957637.10FU	Australia	Soil DNA	-26.52, 151.51	–	Agaricomycetes	975	96.52	0
	KJ188615	SH0957641.10FU	Czechia	*Neottia ovata* mycorrhiza	49.0917, 13.1958	–	Trechisporales	794.7	90.88	0
	KJ188622	SH0957648.10FU	West Bohemia	*Neottia ovata* mycorrhizal seedling (protocorm)	49.0329, 13.4951	–	Agaricomycetes	772.4	90.24	0
	KY574436	SH0957647.10FU	Mexico	*Quercus* ectomycorrhiza	19.981289, -101.197880	–	Agaricomycetes	846.2	90.96	0
	OU953830	SH0957649.10FU	China	Root	–	–	Trechisporales	672.8	90.36	0
	UDB026606	SH0957642.10FU	Morocco	*Cistus creticus* ectomycorrhiza	33.77287002 , -4.98149104	1293	Agaricomycetes	750.1	89.92	0
	UDB042468	SH0957641.10FU	Estonia	Soil DNA	58.4625, 26.72	61	Trechisporales	655.7	89.8	0
	UDB06362957	SH0957649.10FU	Estonia	Soil DNA	–	–	Trechisporales	676.3	90.53	0
	UDB06369764	SH0957646.10FU	Rwanda	Soil DNA	-1.8135, 30.7103	1562	Trechisporales	705.4	91.34	0
	UDB06373747	SH0957641.10FU	Estonia	Soil DNA	58.36329, 26.6557	56	Trechisporales	657.4	89.82	0
	UDB06373749	SH0957641.10FU	Estonia	Soil DNA	58.36329, 26.6557	56	Trechisporales	653.9	89.78	0
	UDB06373751	SH0957641.10FU	Estonia	Soil DNA	58.36329, 26.6557	56	Trechisporales	653.9	89.78	0
	UDB06378154	SH0957641.10FU	Estonia	Soil DNA	58.6612, 25.9695	64	Trechisporales	653.9	89.78	0
	UDB06378158	SH0957641.10FU	Estonia	Soil DNA	58.6612, 25.9695	64	Trechisporales	648.8	89.6	0
	UDB06378162	SH0957641.10FU	Estonia	Soil DNA	58.6612, 25.9695	64	Trechisporales	655.7	89.8	0
	UDB06378352	SH0957641.10FU	Estonia	Soil DNA	58.77773, 25.42577	57	Trechisporales	653.9	89.78	0
	UDB06382819	SH0957642.10FU	Czechia	Soil DNA	50.1899219, 14.8567158	180	Trechisporales	629.9	89.26	0
	UDB06385148	SH0957642.10FU	Germany	Soil DNA	51.42889, 10.02417	285	Trechisporales	629.9	89.26	0
	UDB06385150	SH0957642.10FU	Germany	Soil DNA	51.42889, 10.02417	285	Trechisporales	640.2	89.63	0
	UDB06387915	SH0957642.10FU	Moroco	Soil DNA	32.02366, -6.69507	976	Trechisporales	631.6	89.28	0
	UDB06387917	SH0957642.10FU	Moroco	Soil DNA	32.02366, -6.69507	976	Trechisporales	628.2	89.11	0
	UDB06390280	SH0957641.10FU	Estonia	Soil DNA	58.4625, 26.72	61	Trechisporales	657.4	89.82	0
	UDB06390283	SH0957641.10FU	Estonia	Soil DNA	58.4625, 26.72	61	Trechisporales	653.9	89.78	0
	UDB0647569	SH0957648.10FU	Estonia	Soil DNA	58.42778, 24.28947	14	Trechisporales	633.3	89.05	0
	UDB0703235	SH0957648.10FU	Estonia	Soil DNA	58.25, 27.31666667	–	Trechisporales	633.3	89.05	0
	UDB0750206	SH0957641.10FU	Estonia	Soil DNA	58.384239, 26.721739	–	Fungi	653.9	89.78	0
