# Supplementary material for: Reflexicalyptrales ordo nov., a distinct Agaricomycetes lineage discovered from orchid mycorrhizae
Source: IMA Fungus. 2026 Sep 11;17:e201939. doi: 10.3897/imafungus.17.201939 (PMC13589298; doi:10.3897/imafungus.17.201939)
Supplement: Supplementary file 11 [file imafungus-17-e201939-s016.docx]

**Supplementary References**

Bayev AA, Georgiev OI, Hadjiolov AA et al. (1980) The structure of the yeast ribosomal RNA genes. 2. The nucleotide sequence of the initiation site for ribosomal RNA transcription. Nucleic Acids Research 8(21): 4919–4926. <http://doi.org/10.1093/nar/8.21.4919>

Binder M, Hibbett DS (2002) Higher-level phylogenetic relationships of Homobasidiomycetes (mushroom-forming fungi) inferred from four rDNA regions. Molecular Phylogenetics and Evolution 22(1): 76–90. http://doi.org/10.1006/mpev.2001.1043

Bonito G, Smith ME, Nowak M et al. (2013) Historical biogeography and diversification of truffles in the Tuberaceae and their newly identified southern hemisphere sister lineage. PLoS One 8(1):e52765. <http://doi.org/10.1371/journal.pone.0052765>

Diaz MR, Fell JW (2000) Molecular analyses of the IGS & ITS regions of rDNA of the psychrophilic yeasts in the genus *Mrakia*. Antonie Van Leeuwenhoek. 77(1): 7–12. doi: 10.1023/a:1002048008295

Diederich P, Lawrey JD, Sikaroodi M et al. (2011) A new lichenicolous teleomorph is related to plant pathogens in *Laetisaria* and *Limonomyces* (Basidiomycota, Corticiales). Mycologia 103(3): 525–533. <http://doi.org/10.3852/10-255>

Du XH, Zhao Q, O'Donnell K et al. (2012) Multigene molecular phylogenetics reveals true morels (*Morchella*) are especially species-rich in China. Fungal Genetics and Biology 49(6): 455–469. doi: 10.1016/j.fgb.2012.03.006

Dunham SM, Larsson KH, Spatafora JW (2007) Species richness and community composition of mat-forming ectomycorrhizal fungi in old- and second-growth Douglas-fir forests of the HJ Andrews Experimental Forest, Oregon, USA. Mycorrhiza 17(8): 633–645. <http://doi.org/10.1007/s00572-007-0141-6>

Engel SR, Wong ED, Nash RS et al. (2022) New data and collaborations at the *Saccharomyces* Genome Database: updated reference genome, alleles, and the Alliance of Genome Resources. Genetics 220(4): iyab224. <http://doi.org/10.1093/genetics/iyab224>

Garcia-Sandoval R, Wang Z et al. (2011) Molecular phylogenetics of the Gloeophyllales and relative ages of clades of Agaricomycotina producing a brown rot. Mycologia 103(3): 510–524. <http://doi.org/10.3852/10-209>

Geml J, Davis DD, Geiser DM (2005) Phylogenetic analyses reveal deeply divergent species lineages in the genus *Sphaerobolus* (Phallales: Basidiomycota). Molecular Phylogenetics and Evolution 35(2): 313–22. <http://doi.org/10.1016/j.ympev.2005.01.014>

Hamamoto M, Thanh VN, Nakase T (2002) *Bannoa hahajimensis* gen. nov., sp. nov., and three related anamorphs, *Sporobolomyces bischofiae* sp. nov., *Sporobolomyces ogasawarensis* sp. nov. and *sporobolomyces syzygii* sp. nov., yeasts isolated from plants in Japan.

He MQ, Chen J, Zhou JL et al. (2017) Tropic origins, a dispersal model for saprotrophic mushrooms in *Agaricus* section Minores with descriptions of sixteen new species. Scientific Reports 7(1): 5122. <https://doi.org/10.1038/s41598-017-05203-5>

Hosaka K, Bates ST, Beever Re et al. (2006) Molecular phylogenetics of the gomphoid-phalloid fungi with an establishment of the new subclass Phallomycetidae and two new orders. Mycologia 98(6):949–59. http://doi.org/10.3852/mycologia.98.6.949

Humpert AJ, Muench EL, Giachini AJ et al. (2001) Molecular phylogenetics of *Ramaria* and related genera: evidence from nuclear large subunit and mitochondrial small subunit rDNA sequences. Mycologia, 93(3) 465–477. <https://doi.org/10.1080/00275514.2001.12063180>

Kiyuna T, An K-D, Kigawa R et al. (2015) "Black particles", the major colonizers on the ceiling stone of the stone chamber interior of the Kitora Tumulus, Japan, are the bulbilliferous basidiomycete fungus *Burgoa anomala*. Mycoscience 56: 293–300. https://doi.org/10.1016/j.myc.2014.08.005

Krause C, Garnica S, Bauer R et al. (2011) Aneuraceae (Metzgeriales) and tulasnelloid fungi (Basidiomycota): a model for early steps in fungal symbiosis. Fungal Biology 115(9): 839–851. http://doi.org/10.1016/j.funbio.2011.06.013

Kuuskeri J, Mäkelä MR, Isotalo J et al. (2015) Lignocellulose-converting enzyme activity profiles correlate with molecular systematics and phylogeny grouping in the incoherent genus *Phlebia* (Polyporales, Basidiomycota). BMC Microbiology 15: 217. http://doi.org/10.1186/s12866-015-0538-x

Larsson KH (2007) Re-thinking the classification of corticioid fungi. Mycological Research 111: 1040–1063. http://doi.org/10.1016/j.mycres.2007.08.001

Larsson E, Larsson KH (2003) Phylogenetic relationships of russuloid basidiomycetes with emphasis on aphyllophoralean taxa. Mycologia 95(6):1037–1065. <http://doi.org/10.1080/15572536.2004.11833020>

Lindgren H, Diederich P, Goward T et al. (2015) The phylogenetic analysis of fungi associated with lichenized ascomycete genus *Bryoria* reveals new lineages in the Tremellales including a new species *Tremella huuskonenii* hyperparasitic on *Phacopsis huuskonenii*. Fungal Biology 119(9): 844–56. <http://doi.org/10.1016/j.funbio.2015.06.005>

Liu SL, He SH, Liu DM et al. (2021) Two new species of *Fibrodontia* (Trechisporales, Basidiomycota) with a key to worldwide species. Journal of Fungi 7(11): 982. <http://doi.org/10.3390/jof7110982>

Oberwinkler F, Riess K, Bauer R et al. (2014) Morphology and molecules: the Sebacinales, a case study. Mycological Progress 13: 445–470. <https://doi.org/10.1007/s11557-014-0983-1>

Riess K, Oberwinkler F, Bauer R et al. (2013) High genetic diversity at the regional scale and possible speciation in *Sebacina epigaea* and *S. incrustans*. BMC Evolurionary Biology 13: 102. <http://doi.org/10.1186/1471-2148-13-102>

Riess K, Oberwinkler F, Bauer R et al. (2014) Communities of endophytic sebacinales associated with roots of herbaceous plants in agricultural and grassland ecosystems are dominated by *Serendipita herbamans* sp. nov. PLoS One. 9(4): e94676. <http://doi.org/10.1371/journal.pone.0094676>

Schoch CL, Robbertse B, Robert V et al. (2014) Finding needles in haystacks: linking scientific names, reference specimens and molecular data for Fungi. Database (Oxford) 2014:bau061. <http://doi.org/10.1093/database/bau061>

Skrede I, Engh IB, Binder M et al. (2011) Evolutionary history of Serpulaceae (Basidiomycota): molecular phylogeny, historical biogeography and evidence for a single transition of nutritional mode. BMC Evolutionary Biology 11: 230. <https://doi.org/10.1186/1471-2148-11-230>

Spirin V, Volobuev S, Viner I et al. (2021) On *Sistotremastrum* and similar-looking taxa (Trechisporales, Basidiomycota). Mycological Progress 20: 453–476. <https://doi.org/10.1007/s11557-021-01682-z>

Sulzbacher MA, Baseia IG, Lücking R et al. (2012) Unexpected discovery of a novel basidiolichen in the threatened Caatinga biome of northeastern Brazil. The Bryologist 115(4): 601–609. https://doi.org/10.1639/0007-2745-115.4.601

Sulzbacher MA, Wartchow F, Ovrebo CL et al. (2016) *Sulzbacheromyces caatingae*: notes on its systematics, morphology and distribution based on ITS barcoding sequences. The Lichenologist 48(1): 61–70. doi:10.1017/S0024282915000420

Tedersoo L, Bahram M, Ryberg M et al. (2014) Global biogeography of the ectomycorrhizal /sebacina lineage (Fungi, Sebacinales) as revealed from comparative phylogenetic analyses. Molecular Ecology 23: 4168–4183. <http://doi.org/10.1111/mec.12849>

Trierveiler-Pereira L, da Silveira RM, Hosaka K (2014) Multigene phylogeny of the Phallales (Phallomycetidae, Agaricomycetes) focusing on some previously unrepresented genera. Mycologia 106(5): 904–911. <http://doi.org/10.3852/13-188>

Uehling JK, Henkel TW, Aime MC et al. (2012) New species of *Clavulina* (Cantharellales, Basidiomycota) with resupinate and effused basidiomata from the Guiana Shield. Mycologia 104(2):547–556. <http://doi.org/10.3852/11-130>

Vu D, Groenewald M, Szöke S et al. (2016) DNA barcoding analysis of more than 9000 yeast isolates contributes to quantitative thresholds for yeast species and genera delimitation. Studies in Mycology 85: 91–105. http://doi.org/10.1016/j.simyco.2016.11.007

Wang S, Thorn RG (2021) *Exidia qinghaiensis*, a new species from China. Mycoscience 62(3): 212–216. http://doi.org/10.47371/mycosci.2021.03.002

Wu F, Tohtirjap A, Fan L-F et al. (2021) Global diversity and updated phylogeny of *Auricularia* (Auriculariales, Basidiomycota). Journal of Fungi 7(11): 933. <https://doi.org/10.3390/jof7110933>

Zamora JC, Ekman S (2020) Phylogeny and character evolution in the Dacrymycetes, and systematics of Unilacrymaceae and Dacryonaemataceae fam. nov. Persoonia 44: 161-205. <http://doi.org/10.3767/persoonia.2020.44.07>

Zhang R, Shi XF, Liu PG et al. (2022) Host shift speciation of the ectomycorrhizal genus *Suillus* (Suillineae, Boletales) and biogeographic comparison with its host Pinaceae. Frontiers in Microbiology 30:831450. doi: 10.3389/fmicb.2022.831450
